# Regulation of the Phase Structure in the Crystallizing Curing System PCL–DGEBA

**DOI:** 10.3390/polym16192695

**Published:** 2024-09-24

**Authors:** Irina O. Plyusnina, Uliana V. Nikulova, Ramil R. Khasbiullin, Aleksey V. Shapagin

**Affiliations:** Frumkin Institute of Physical Chemistry and Electrochemistry Russian Academy of Sciences (IPCE RAS), 31, Building 4 Leninsky Prospect, Moscow 119071, Russia; irinaplyusninar@yandex.ru (I.O.P.); ulianan@rambler.ru (U.V.N.); khasbiullin@techno-poisk.ru (R.R.K.)

**Keywords:** epoxy resin, DGEBA, polycaprolactone, phase diagram, phase reversal region, interpenetrating phases, phase structure, interdiffusion

## Abstract

Qualitative and quantitative aspects of the formation of various types of phase structures, sizes and compositions were considered. For the studied polycaprolactone–epoxy resin/4,4′-diaminediphenylsulfone system, a phase diagram characterized by amorphous separation with a lower critical solution temperature was constructed and its evolution was traced with increasing conversion degree of epoxy groups. A method is proposed for determining the temperature–concentration parameters that determine the type of phase structure of composite materials, based on the optical interferometry method. All types of phase structures and features of structure formation in the phase reversal region and at its boundaries have been studied using optical and scanning electron microscopy methods. The dimensions of the structural elements were determined and their correlation with the temperature and concentration regimes of the system’s curing was established. The composition of phases in cured compositions was studied using FTIR spectroscopy, DSC and scanning electron microscopy. It is shown that by varying the temperature–concentration parameters of curing reactive thermoplastic systems, it is possible to specifically regulate the type of phase structure, phase sizes and their composition, which determine the operational properties of the material.

## 1. Introduction

Epoxy resins are one of the important polymers that are widely used in various fields of technology due to their manufacturability and good performance properties, as well as low cost. However, epoxy resins have limitations in their use due to the disadvantage of fragility [1,2]. One of the key strategies for improving the stress–strength properties of thermoset materials is their modification with components with a low elastic modulus, for example, rubbers and thermoplastics [3]. Recently, most works have been devoted to the modification of epoxy resins with rigid-chain thermoplastics, which is explained by their higher thermophysical properties [4,5,6]. Most uncured thermoset/thermoplastic mixtures are homogeneous and form a heterogeneous structure when cured [7,8]. A significant number of studies have shown that, due to the phase structure of thermoplastic-modified epoxy systems formed during curing, composites can surpass homopolymers in a whole range of characteristics [9,10].

An analysis of the literature showed that the bulk of researchers are focused on studying the correlation between the properties and concentration of the modifier in the mixture [11,12]. While the operational characteristics are affected not so much by the concentration of the modifier, but by the type of phase structure and the size of the structural elements [13,14]. In a curing multicomponent system, phase decomposition of various types can occur, as a result of which the following types of phase structures will be formed in the material:

- Matrix dispersion, where the matrix is enriched with an epoxy component;
- Interpenetrating phases (IP);
- Inverted matrix dispersion, where the matrix is enriched with a thermoplastic component.

Although many studies have explored the phase structures of epoxy resins modified with thermoplastics, few have focused on the detailed relationship between curing parameters (temperature and concentration) and phase structure, particularly in the phase reversal region.

Thus, it is of interest to study the physicochemical and technological parameters that make it possible to regulate the structure of cured systems and, as a consequence, their properties.

The most informative approach to predicting the phase structure is to construct phase diagrams over a wide range of temperatures and mixture compositions. This information makes it possible to qualitatively and quantitatively describe structural transformations during the curing reaction and predict the phase organization in the system in various regions of the temperature–concentration field of the phase diagram.

In our opinion, the most interesting zone of the phase diagram is the phase reversal region characterized by the phase structure of the interpenetrating phase type (IP-type), which has received little attention in the literature due to its localization in a narrow concentration range. This region separates zones in the phase diagram corresponding to matrix-dispersion structures. A material characterized by this type of phase structure is a combination of the properties of an epoxy resin and a modifier [15].

The development of a quantitative method for determining temperature and concentration parameters that determine the type of phase structure of a cured composition is of fundamental importance for predicting the performance characteristics of a composite material. In our opinion, by finding the concentration point corresponding to the center of a narrow phase reversal region, it is possible to quantitatively predict the type of phase structure and, accordingly, the properties of the material corresponding to one or another type of phase structure. When studying the physical and mechanical characteristics of a material, it is also important to predict the composition and size of phases. Using crack resistance as an example, it could be predicted that the size of the phases directly affects the resistance of the system to crack propagation, and the composition of the phase directly affects the fracture mechanism. In the case of systems capable of self-healing, the size of the dispersed phase is significant since the thermoplastic-enriched phase acts as a reservoir of self-healing agents to fill the crack [16,17].

The epoxy resin (DGEBA)–polycaprolactone (PCL) system was chosen as a model system that allows us to establish a set of physicochemical and technological parameters and their influence on the phase structure, composition and dimensions of structural elements. PCL is a partially crystalline biodegradable polymer and has a low melting point of about 63 °C. Sufficiently high diffusion coefficients make it possible to create model systems characterized by different types of phase structures and wide ranges of phase sizes and compositions. It is known from the literature that materials based on epoxy resin modified with PCL due to its melting and crystallization are characterized by self-healing properties [18].

Note that the morphology of the cured PCL–DGEBA system has been widely studied in the literature. However, in all studies, the authors mixed epoxy resin with PCL with a certain concentration step, after which they studied the structure and properties [16,19,20]. We propose a different approach to study the phase structure of curing modified systems: prediction of the structure of a composite material cured at a given temperature. As a result of one experiment, we determine the temperature and concentration parameters of all types of phase structures of the studied system. This approach allows us not only to create materials with the required properties, but also to significantly reduce the time for creating a material with a given phase structure.

This work is a continuation of our previously published work [21] and consists of a comprehensive study of structure formation in the crystallizing reactive composition PCL–DGEBA, cured with DDS. In this regard, the article presents the results of studies of parameters that determine the type of phase structure, size and composition of coexisting phases.

The fundamental research presented in this article brings us closer to solving the applied problem of developing a tool that allows, without resorting to routine screening studies, having physicochemical data on the components and their mixing in the initial state and at different stages of the chemical reaction of curing, to obtain information for technologists about the concentrations of components and technological parameters for obtaining a material characterized by the required phase structure, providing the necessary performance properties.

## 2. Materials and Methods

### 2.1. Materials

**Figure 1:**
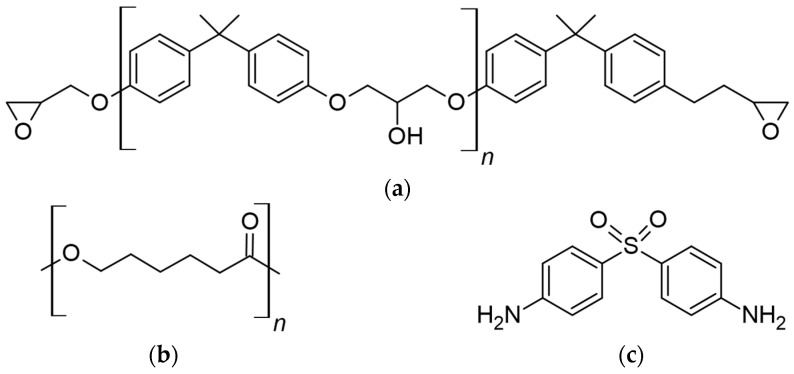
Chemical structures: (**a**) DGEBA, (**b**) PCL and (**c**) DDS.

Epoxy oligomer DGEBA brand KER 828 (KUMHO P&B Chemicals, Gwangju, South Korea) is a low-molecular-weight liquid polycondensation product of epichlorohydrin with bisphenol A with Epoxide Equivalent Weight (EEW) 188.5 g/eq. Polycaprolactone PCL (“Sigma-Aldrich”, Darmstadt, Germany) has a molecular weight M_n_ = 45 × 10^3^ g/mol, ρ = 1.145 g/cm^3^. The aromatic amine 4,4′-diaminediphenylsulfone (DDS) brand Aradur 9664-1 (Huntsman International LLC, The Woodlands, TX, USA) was used as a hardener. The structural chemical formulae of DGEBA, PCL and DDS are shown in Figure 1. PCL with epoxy oligomer adducts (lower DDS content in DGEBA relative to the stoichiometric ratio) was also studied.

### 2.2. Methods

#### 2.2.1. Optical Interferometry (OI)

**Figure 2:**
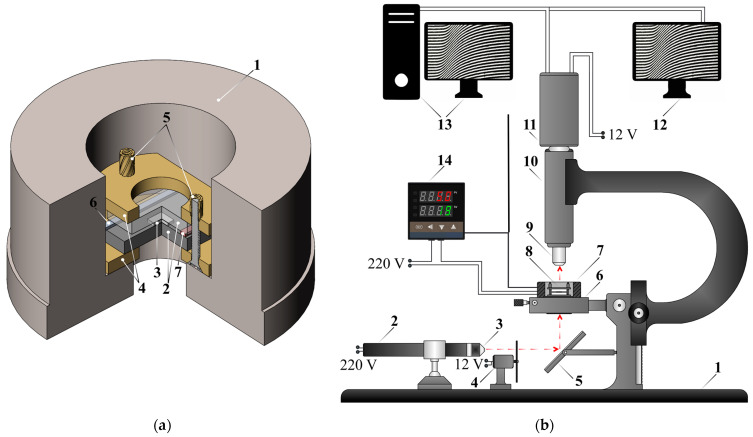
(**a**) Scheme of a diffusion cuvette and cell: 1—thermostatic shell, 2—glass plates, 3—sample, 4—cell clamps, 5—screws, 6,7—wedges of different thicknesses; (**b**) scheme of the ODA-2 optical interferometer: 1—frame, 2—helium-neon laser, 3—beam expander, 4—optical diffuser, 5—metal mirror, 6—goniometric table, 7—diffusion cuvette, 8—diffusion cell, 9—optical microscope lens, 10—magnification tube, 11—video camera, 12—auxiliary monitor, 13—computer, 14—thermostatting system. The arrows show the beam path.

Using the method of optical interferometry on an ODA-2 interferometer (IPCE RAS, Moscow, Russia), the scheme of which is shown in Figure 2, phase equilibria in the PCL–DGEBA system in the presence of DDS were studied in order to construct phase diagrams at different stages of the chemical curing reaction and determine concentration ranges, characterized by different types of phase structures.

The studies were carried out at temperatures from 140 to 200 °C. DGEBA was first mixed with DDS in a ratio of 10:3 at 90 °C at a speed of 1200 rpm for 1 h until DDS was completely dissolved. All studies were carried out on PCL films with a thickness of ~150 μm, obtained by pressing at 120 °C. The PCL film was placed in a diffusion cell between glasses whose inner sides were coated with a translucent layer of Ni-Cr alloy. An angle of ≤2° was set between the glasses, which was necessary for the appearance of interference pictures when passing a laser beam with λ = 635 nm. After assembly, the cell was thermostated at the given experimental temperature for at least 30 min. Then, the space between the glasses was filled with a mixture of DGEBA + DDS or an adduct (aDGEBA). The moment of contact of the components was considered the beginning of the process of interdiffusion. After some time, the beginning of the formation of a phase boundary was observed. At this moment, interferograms were recorded to determine the concentration point and the time of onset of phase decomposition.

When working with adducts, after coupling the components, the system was thermostated until the interference pictures were completely stabilized. To construct a phase diagram, the system was scanned at temperatures in the range of 140 to 200 °C with a step of 5 °C in the heating–cooling mode. At each stage, the system of conjugate phases was thermostated for at least 30 min. After the appearance of the phase boundary and holding the system to an equilibrium state, interferograms were recorded and the compositions of the coexisting phases were determined.

The methods for processing the diffusion zones of interferograms and construction phase diagrams did not differ from those described previously [22,23] and are presented below.

#### 2.2.2. Differential Scanning Calorimetry (DSC)

Using a Netzsch DSC 204F1 Phoenix differential scanning calorimeter (Netzsch-Gerätebau GmbH, Selb, Germany), kinetic dependences of the degree of curing were obtained in isothermal modes at 150, 180 and 200 °C for PCL–DGEBA + DDS mixtures. The values of the melting temperatures of the phases of heterogeneous mixtures PCL–DGEBA + DDS with a PCL content of 23, 28 and 38 vol.% cured at 150 °C and 20, 24 and 38 vol.% cured at 200 °C were also determined. The scanning speed in all experiments was 10 °C/min. Sample preparation did not differ from traditional [24].

#### 2.2.3. Fourier Transform Infrared Spectroscopy (FTIR)

IR spectra of PCL, DGEBA + DDS and cured PCL–DGEBA + DDS samples, as well as spectral maps of the cured PCL–DGEBA + DDS mixture, were obtained using a Nicolet iN10 IR microscope (Thermo Fisher Scientific, Waltham, MA, USA) at spectral range of 4000–675 cm^−1^. The spectra were recorded on a germanium crystal in ATR mode as an average of 128 scans at a resolution of 4 cm^−1^. Spectral processing was performed using Omnic 9.2.86 software (Thermo Fisher Scientific, Waltham, MA, USA). When taking the IR spectrum at a point, the aperture was 100/100 µm. To identify the phases in the cured PCL–DGEBA + DDS mixture, IR spectral maps were obtained with apertures of 50/50. For each individual spectrum and the spectrum at each map point, automatic baseline correction was performed. For quantitative calculations, the intensities of individual bands were normalized by the intensity of the stretching vibration band of the CH_2_ group (2870 cm^−1^).

#### 2.2.4. Scanning Electron Microscopy (SEM) with EDX

Studies of the phase structure and determination of the elemental composition of the studied objects were carried out using a JSM-6060A scanning electron microscope (JEOL, Japan). To prepare the samples, DGEBA was mixed with DDS in a ratio of 10:3 at a temperature of 90 °C at a speed of 1200 rpm until a homogeneous solution was obtained. The required amount of PCL was then added to obtain the systems presented in Table 1. The mixture was homogenized, poured into a silicone mold, degassed and cured at temperatures of 150, 180 and 200 °C until complete conversion of the epoxy groups. The phase structure of sample chips was revealed by etching in a low-frequency oxygen discharge plasma on a universal vacuum station (Edwards Coating System E306A, Edwards Vacuum, Burgess Hill, UK) for 40 min. Preparation consisted of vacuum thermal spraying of a conductive carbon layer onto the surface of a sample of the cured system in a DSR device (Nano-Structured Coatings Co., Tehran, Iran). SEM images of the sample were obtained in secondary electrons at an accelerating voltage of 15 keV. Characteristic X-ray spectra were obtained at points with a collection time of 120 s. (SEM photos in their original size are presented in the Supplementary Materials).

#### 2.2.5. Optical Microscopy (OM)

The formation of phase structures in situ of a mixture of PCL–DGEBA + DDS at T = 200 °C with a PCL content of 22, 24, 26 vol.% was observed on an Olympus BX 51 optical microscope (Olympus, Tokyo, Japan). To prepare the samples, DGEBA was mixed with DDS in a ratio of 10:3 at a temperature of 90 °C at a speed of 1200 rpm until a homogeneous solution was obtained. Then, the required amount of PCL was added and homogenized. The melt sample was applied to a glass slide and placed on a thermostated microscope stage (Linkam LTS350, Linkam Scientific Instruments Ltd, Tadworth, Surrey, UK) at the experimental temperature.

### 2.3. The Studied Systems

PCL–DGEBA + DDS systems with different concentrations of components were used to conduct in in situ studies during the curing process and analyze the final mixtures. Temperature conditions and methods for studied systems are presented in Table 1.

## 3. Results and Discussions

The physical, mechanical and other operational characteristics of curing multicomponent materials are influenced by the phase structure formed during the curing reaction. In this regard, we divided the results obtained in the work into several parts, in which we consider the influence of technological and physicochemical parameters on the type of phase structure, the size of structural elements and the composition of coexisting phases.

### 3.1. Prediction of the Phase Structure Type

The optical interferometry method allows us to study, in situ, phase transformations over the entire concentration range at a given temperature during the curing process. In this work, we first tried to obtain information about the concentration range corresponding to the phase reversal region of the phase diagram. This was based on the principle of evolution of the phase diagram during the curing reaction, shown in the scientific literature and in our previous works [25]. It is known that, as the degree of conversion increases, the viscosity of the system increases and the thermodynamic compatibility of the components deteriorates. This is accompanied by an expansion of the binodal curve of the phase diagram along the scale of concentrations and temperatures. In the case of systems characterized by an upper critical solution temperature (UCST), the critical temperature increases and for systems with a lower critical solution temperature (LCST), it decreases. Thus, with the time of formation of the diffusion zone in the PCL–DGEBA + DDS system in the isothermal mode, after some time, the binodal dome crosses the isotherm and a phase boundary appears on the interferogram of the diffusion zone. It is important that it is localized in the concentration region corresponding to the critical concentration at the moment the isotherm is crossed by the binodal dome. Thus, we determine the concentration of the mixture, the curing of which at a given temperature (a figurative point on the temperature–concentration field of the phase diagram) leads it to fall directly into the middle of the phase reversal region of the binodal curve of the phase diagram. Systems whose figurative points fall into the region of phase reversal after curing are characterized by an interpenetrating phase (IP)-type structure.

**Figure 3:**
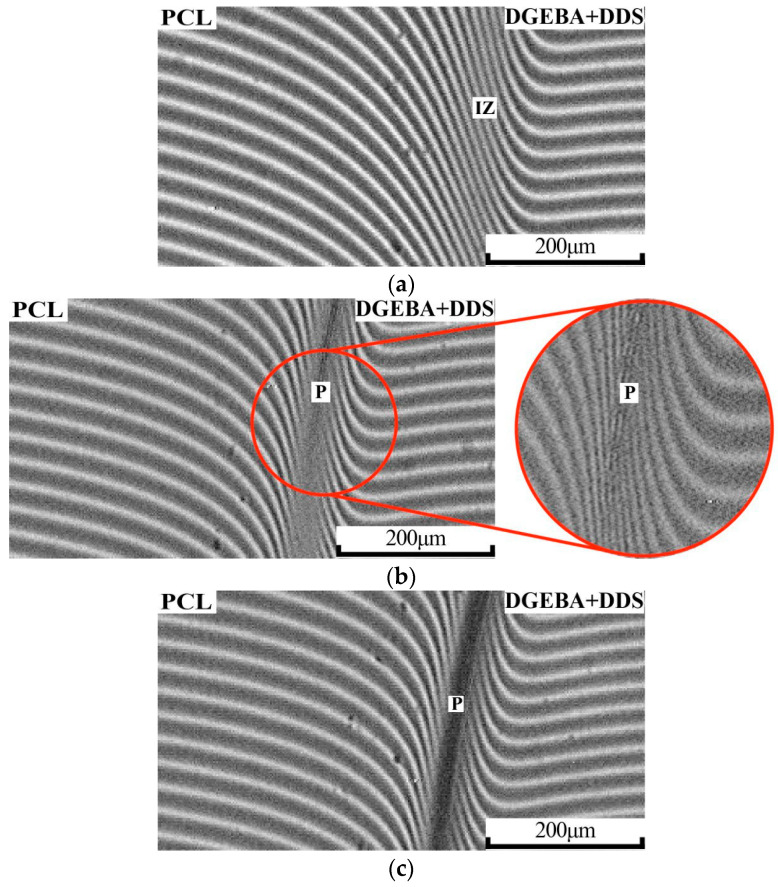
Interferograms of the PCL–DGEBA + DDS system at T = 150 °C and time: (**a**) 25, (**b**) 87, (**c**) 94 min, where P is the phase boundary, IZ is the interdiffusion zone.

Figure 3 shows interferograms of the diffusion zone of the conjugate phases PCL and DGEBA + DDS at 150 °C.

It has been shown that, at short curing times, corresponding to low degrees of conversion, gradient homogeneous solutions are formed in the transition region (Figure 3a). With further curing of the PCL–DGEBA + DDS system (Figure 3b), the appearance of a phase boundary (P) was observed in the interdiffusion zone (IZ). At the experimental temperature, the calculated concentration of PCL in the PCL–DGEBA + DDS mixture, corresponding to the center of the phase reversal region and the emergence of a type of IP phase structure, is 28 vol.% The curing process is accompanied by a broadening of the heterogeneous region in the interferogram (Figure 3c), which is reflected in the expansion of the binodal curve of the phase diagram. Note that with an increasing degree of conversion, the critical concentration of the curing system shifts towards the thermoplastic in accordance with the classical Flory–Huggins theory of polymer solutions. As a result, the concentration zone of phase reversal also shifts to a region enriched in PCL, which, in the case of high diffusive mobility of the system, can lead to a change in the type of phase organization. It is important that the processes of changing the phase structure type from nucleated to final (cured) are realized only under conditions of good macromolecular mobility.

The studies were carried out at temperatures of 180 and 200 °C. For these temperatures, the concentrations of PCL in the PCL–DGEBA + DDS mixture, characterized by the IP type structure, were 26 and 24 vol.%, respectively.

**Figure 4:**
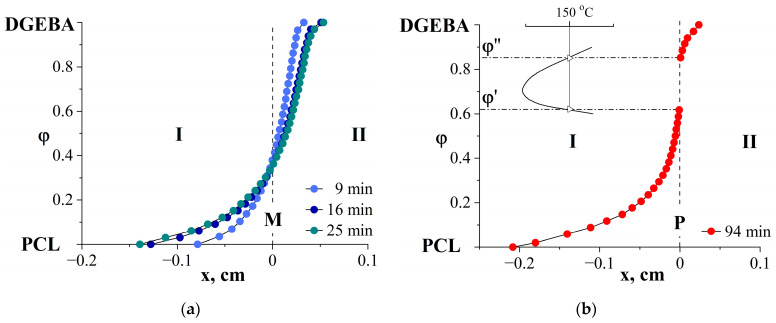
Concentration distribution profiles in the PCL–DGEBA + DDS system at 150 °C. Diffusion and curing time: (**a**) 9, 16, 25 min; (**b**) 94 min. I corresponds to a diffusion of DGEBA + DDS in PCL, II is a diffusion of PCL in DGEBA + DDS. M is Matano–Boltzmann plane, φ′ and φ′′ are compositions of coexisting phases.

To quantitatively describe the curing process, concentration distribution profiles in diffusion zones at different stages of the chemical reaction were constructed from interference pictures. Typical profiles are shown in Figure 4.

At the initial stage of curing, before the process isotherm crosses the binodal dome, it can be seen that the system is characterized by continuous *S*-shaped concentration curves showing changes in the compositions of gradient solutions (Figure 4a). The position of the Matano–Boltzmann plane, corresponding to the concentration at which the diffusion fluxes of the components into each other are equal, is constant and corresponds to the average composition region.

There is an overlap of concentration dependences for 16 and 25 min of curing (Figure 4a), which is explained by a decrease in interdiffusion coefficients and the system reaching the gelation point. Subsequent processes, as noted above, lead to the appearance of a phase boundary, at which a concentration jump from φ′ to φ″ occurs (Figure 4b). These concentrations correspond to the compositions of the coexisting phases of the binodal curve of the phase diagram.

**Figure 5:**
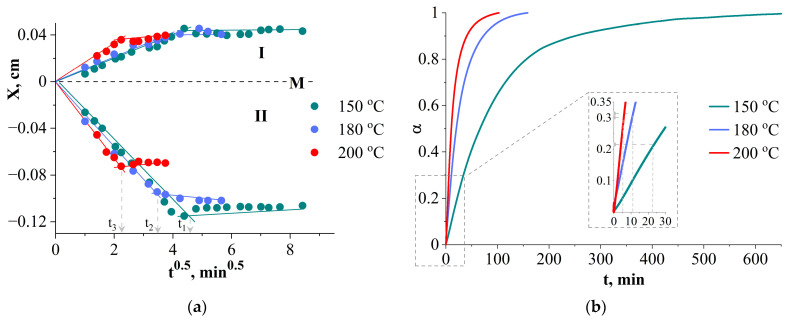
Kinetic dependencies: (**a**) movements of isoconcentration planes; (**b**) conversion rate at φPCL 28 vol.% curing mixture PCL–DGEBA + DDS. Diffusion zones: I—PCL in DGEBA + DDS, II—DGEBA + DDS in PCL. M is Matano–Boltzmann plane.

Measurements of diffusion constants during the mutual dissolution of components, as well as their changes during the chemical reaction, were calculated from interference pictures when the systems were kept in isothermal mode. It has been established that the nature of the kinetic dependences of the movement of isoconcentration planes at the initial stages of mixing (Figure 5a) is linear, which indicates the diffusion mechanism of components mixing in the selected temperature range from 150 to 200 °C. During the chemical reaction of curing, the mass transfer rates slow down, which is expressed in a change in the slope angles of the dependences presented in Figure 5a.

For the temperature–time intervals in which the diffusion mixing mechanism was observed, the values of the diffusion coefficients for the maximum concentrations of the systems were calculated. It has been established that the system is characterized by fairly high diffusion mobility (10^−7^ cm^2^s^−1^ diffusion of PCL in DGEBA + DDS and 10^−6^ cm^2^s^−1^ diffusion of DGEBA + DDS in PCL).

The kinetic curves of the degree of conversion were obtained using the DSC method at temperatures of 150, 180 and 200 °C (Figure 5b). The curves have a general tendency for the degree of curing to vary with temperature. The rate of reaction increases with increasing temperature. Thus, at a higher experimental temperature, less reaction time is required to achieve the same degree of conversion. A joint analysis of the dependencies in Figure 5 shows that the degree of curing at the gelation point varies in the range from 0.21 to 0.31 (Table 2).

**Figure 6:**
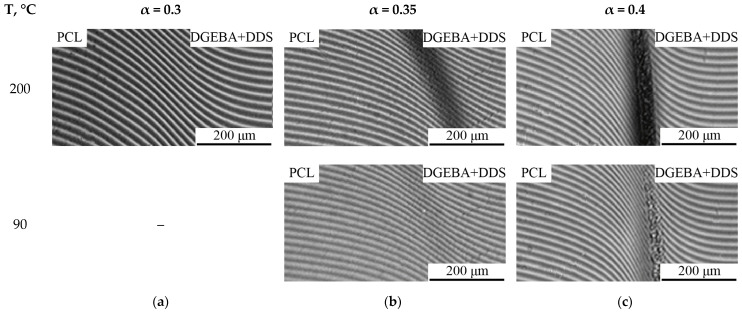
Interferograms of interdiffusion zones of the PCL–aDGEBA system at 200 and 90 °C. Conversion rate: (**a**) 0.3; (**b**) 0.35; (**c**) 0.4.

Note that at high temperatures (180 and 200 °C), the curing process occurs at a high speed; therefore, the conversion rates at the gelation point for these temperatures are close. Also, in accordance with the experimental data obtained, the formation of heterogeneous structures occurs after the system reaches the gelation point t_pd_ ≥ t_gel_, which correlates with data from the literature [26]. For this purpose, and in order to determine the type of amorphous separation phase diagram (UCST or LCST), additional studies of the mixtures involved mixing PCL with DGEBA α ≥ 0.3 adducts simulating various stages of curing, in heating and cooling cycles. The adducts were cured during the experiment. Figure 6 shows interferograms of interdiffusion zones when PCL is coupled with aDGEBA.

In the interdiffusion zone at α = 0.3 (Figure 6a), the system is homogeneous, which correlates with the obtained data presented above. At 200 °C α = 0.35 (Figure 6b), a phase boundary is formed in the interdiffusion zone, and with decreasing temperature, the phase boundary disappears, and the gradient zone is characterized by a continuous concentration profile. Note that this trend is reproducible in heating–cooling cycles for a system with α = 0.35 and it can be argued that the system is characterized by amorphous stratification with LCST. In the case of α = 0.4 (Figure 6c), a phase boundary is formed and remains in the same position as the temperature decreases; only the concentration jump, corresponding to the narrowing of the heterogeneous region of the phase diagram, decreases in the concentration profile. Such changes in the system are due to a significant decrease in diffusion coefficients when passing through the gelation point (Figure 5a) as a result of the formation of a dense network of chemical bonds, which prevents the dissolution of thermoplastic macromolecules in aDGEBA.

**Figure 7:**
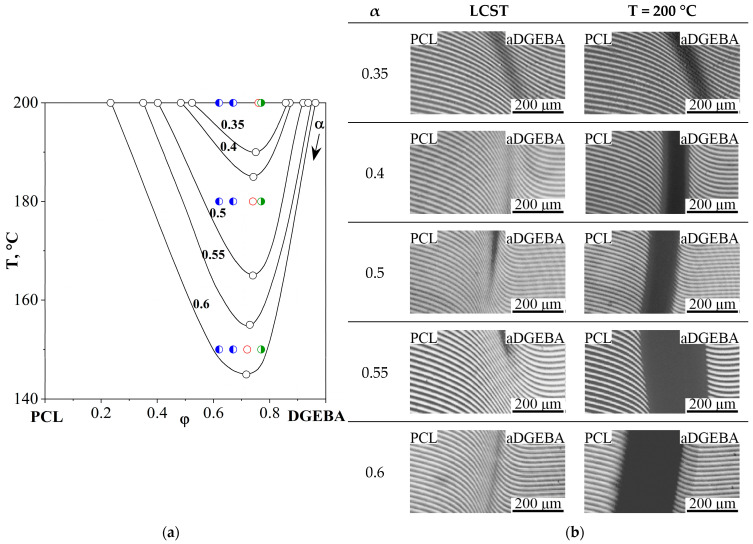
(**a**) Evolution of the phase diagram of the PCL–aDGEBA system during the chemical curing reaction. The conversion rates are shown near the corresponding binodal curves; (**b**) interferograms of the PCL–aDGEBA system. The dots indicate the compositions of the mixtures: 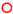 determined by optical interferometry; 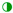 (23 vol.%) and 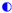 (33, 38 vol.%) are characterized by matrix dispersion and matrix-dispersion structures with an inverted phase structure.

In thermoplastic–DGEBA systems, the distance of the critical temperature of the initial system in the temperature–concentration field of the phase diagram from the isotherm of the curing process determines at what degree of conversion of epoxy groups phase decomposition will begin. In order to determine the temperature dependences of the critical parameters of the curing system (α^cr^ and φ^cr^), the solubility of adducts with degrees of curing from 0.3 to 0.6 in PCL was studied over the entire concentration range at temperatures from 150 to 200 °C. Phase diagrams of amorphous separation at different stages of the chemical curing reaction were constructed (Figure 7). Temperature limitations when studying the evolution of the phase diagram are due to the fact that systems with α ≤ 0.3 are characterized by phase diagrams with LCST > 200 °C, located in the region of thermal destruction of PCL [27,28], and, at T < 140 °C, the rate of the chemical reaction of curing is extremely small.

**Figure 8:**
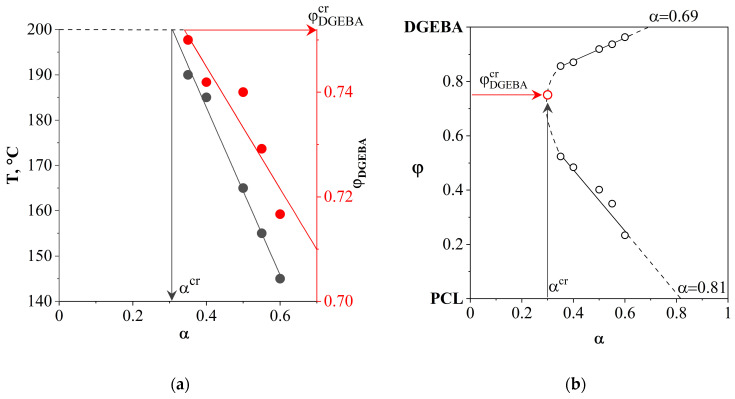
(**a**) Dependencies of critical temperatures and DGEBA content on the degree of conversion; (**b**) isothermal cross-section of phase diagrams at various degrees of conversion. T = 200 °C.

By scanning the PCL–aDGEBA system by temperature, we recorded the appearance of a phase boundary on the concentration profile (Figure 7b), the position of which corresponds to the critical concentration of the binodal dome in the phase diagram (Figure 7a). When the experimental temperature was increased to 200 °C, an expansion of the heterogeneous region was observed and the compositions of the coexisting phases were determined. It has been established that an increase in the degree of curing contributes to the broadening of the heterogeneous region and the shift of the LCST to the region of lower temperatures. From the obtained phase diagram (Figure 7a), the values of critical temperatures, concentrations and degrees of conversion are presented in Figure 8a.

The resulting linear dependencies make it possible to determine the critical concentration and degree of conversion at the selected curing temperature of the system. Thus, we determine the concentration of the modifier to obtain cured systems characterized by the IP-type structure. We also determine the degree of conversion of the onset of phase decomposition, which affects the diffusion constants of mass transfer, which determines the size of the final phase structures.

Figure 8b presents data obtained from an isothermal cross-section of phase diagrams at various degrees of conversion (T = 200 °C). The critical parameters of phase decomposition (α^cr^ = 0.3 and φ^cr^_DGEBA_ = 0.751) at T = 200 °C were determined and it was found that, at a conversion degree of 0.81, the composition of the coexisting phases should correspond to pure components.

**Figure 9:**
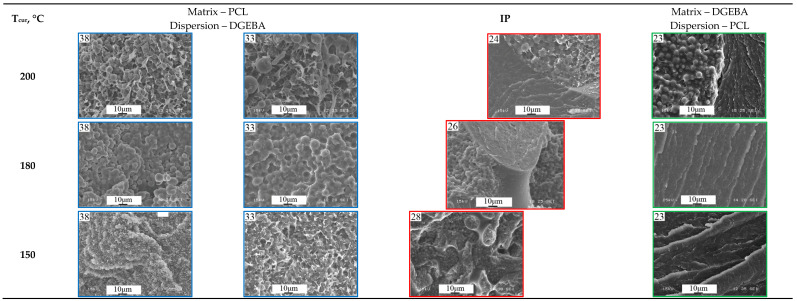
SEM images of the phase structure of cured PCL–DGEBA + DDS systems at temperatures: 150, 180 and 200 °C. The PCL contents in the mixtures are indicated in the pictures (SEM photos in their original size are presented in the Supplementary Materials).

The phase diagram of the evolution of phase states during the curing process (Figure 7a) shows the compositions of mixtures determined by optical interferometry for the phase reversal region at temperatures of 150, 180 and 200 °C. To validate the proposed method for determining the critical concentration that determines the structure of the IP type in reactive thermoplastic systems, the structure of cured systems was studied using SEM. Additionally, the structure of systems containing PCL contents of 33, 38 and 23 vol.% was studied at the same temperatures in order to confirm that these systems are characterized by matrix dispersion and matrix-dispersion structures with an inverted phase structure, respectively. These systems also allowed us to analyze the effect of curing temperature on the size of phase structures. SEM images of the cured compositions are presented at a single magnification in Figure 9.

**Figure 10:**
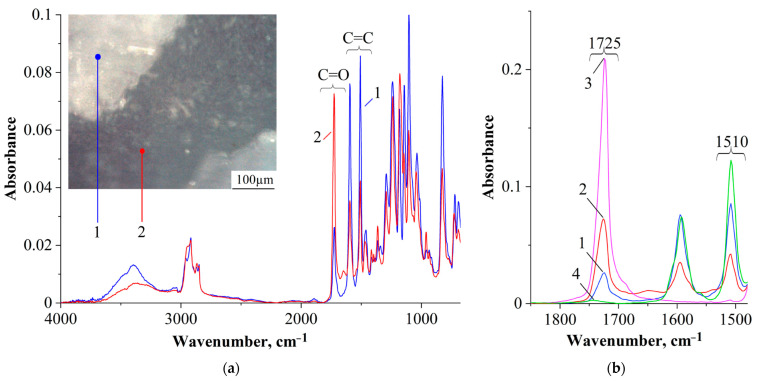
(**a**) FTIR spectrum of the PCL–DGEBA + DDS system cured at 200 °C with a PCL content of 24 vol.%; (**b**) fragment of the FTIR spectrum 1—light region, 2—dark region, 3—spectrum of pure PCL, 4—DGEBA spectrum of cured DDS.

**Figure 11:**
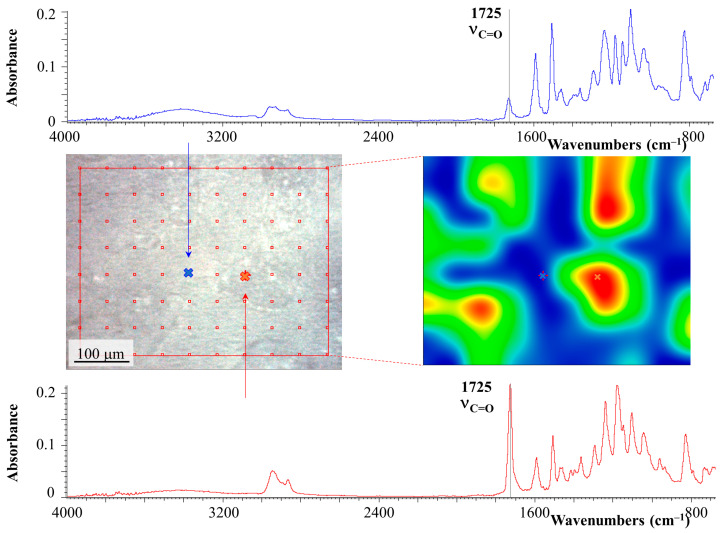
FTIR spectra and spectral map of a PCL–DGEBA + DDS mixture cured at 180 °C with a PCL content of 26 vol.%.

**Figure 12:**
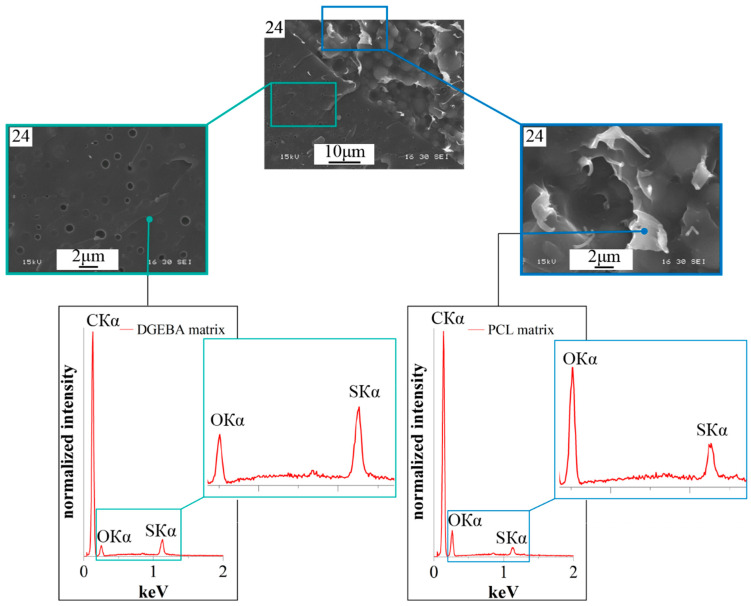
SEM image of the phase structure of the cured PCL–DGEBA + DDS system at 200 °C with a PCL content of 24 vol.% and EDX spectra from the area.

It can be seen that all the studied mixtures are characterized by a heterogeneous phase structure. As expected, the structure of mixtures with component concentrations determined by optical interferometry, formed as a result of curing, is characterized by the IP type. It is important that the ratio of the volume fractions of continuous phases to each other is ~50:50. This suggests that certain mixture concentrations actually correspond to the center of the phase reversal concentration region in the phase diagram. Qualitative identification of the compositions of coexisting phases of PCL–DGEBA + DDS mixtures, characterized by the IP-type structure, was carried out on a system with a PCL content of 24 vol.% (Figure 9) using FTIR spectroscopy (Figure 10 and Figure 11) and EDX (Figure 12).

In a preliminary experiment, FTIR spectra of PCL and DGEBA of cured DDS were obtained and analyzed, fragments of which are presented in Figure 10b (spectra 3 and 4, respectively). These spectra correlate well with the standard spectra of these polymers and contain characteristic absorption bands for the main bond groups. Particular attention was paid to those absorption bands that were absent in the FTIR spectrum of one polymer and present in the other. For example, PCL is characterized by the presence of a carbonyl group C=O and a corresponding very intense absorption band at 1725 cm^−1^. At the same time, the FTIR spectrum of DGEBA + DDS is characterized by the presence of double bonds C=C of the aromatic ring and corresponding absorption bands at 1596 and 1510 cm^−1^. Figure 10a shows an optical microphoto of the PCL–DGEBA + DDS system with a PCL content of 24 vol.%, obtained on an FTIR microscope with two fragments of continuous phases and the corresponding FTIR spectra (blue spectrum 1 for the light region and red spectrum 2 for the dark). It is clearly seen that both spectra of the mixture (spectra 1 and 2) contain both absorption bands of the carbonyl group of PCL and absorption bands of the double bond of the aromatic ring of DGEBA. But the intensities of these bands vary greatly for different regions. Thus, if the dark region (spectrum 2) is predominantly represented by a high-intensity band at 1725 cm^−1^ and low-intensity bands in the range of 1600–1500 cm^−1^, then the light region (spectrum 1), on the contrary, has a low peak intensity at 1725 cm^−1^ and is noticeably larger at 1596 and 1510 cm^−1^. This suggests that the dark region is enriched in PCL, while the light region, on the contrary, is depleted of it.

In addition to identifying the qualitative composition of coexisting phases in the region of phase inversion, a spectral map of the distribution of characteristic radiation corresponding to the stretching vibrations of the carbonyl group C=O of PCL (1725 cm^−1^) was obtained (Figure 11).

It can be seen that the continuous phase, indicated in blue, has a low concentration of carbonyl groups, which indicates that it is enriched in epoxide. Note that it does not identify the dispersed phase enriched in PCL, determined by SEM, due to its small size. The second continuous phase, on the contrary, is enriched in PCL and has a wide distribution of its concentrations (color reflecting the intensity of the characteristic radiation, from green to red). This confirms that the composition of this phase is characterized by the left branch of the binodal (Figure 7) and includes a matrix enriched in PCL and large dispersed phases enriched in epoxide.

Identification of the qualitative composition of coexisting continuous phases was also carried out using EDX X-ray microanalysis (Figure 12) based on the ratio of the intensities of the characteristic Kα lines of oxygen (OKα) and sulfur (SKα). It can be seen that the continuous phase with dispersed structure sizes of no more than 1 μm is enriched in epoxide since the intensity of the sulfur Kα line exceeds the oxygen line. On the contrary, the matrix of the phase with dispersed particles larger than 3 μm is enriched in PCL since the intensity of the sulfur Kα line is several times lower than the intensity of the oxygen line.

### 3.2. Evolution of Phase Morphology in the Phase Reversal Region

**Figure 13:**
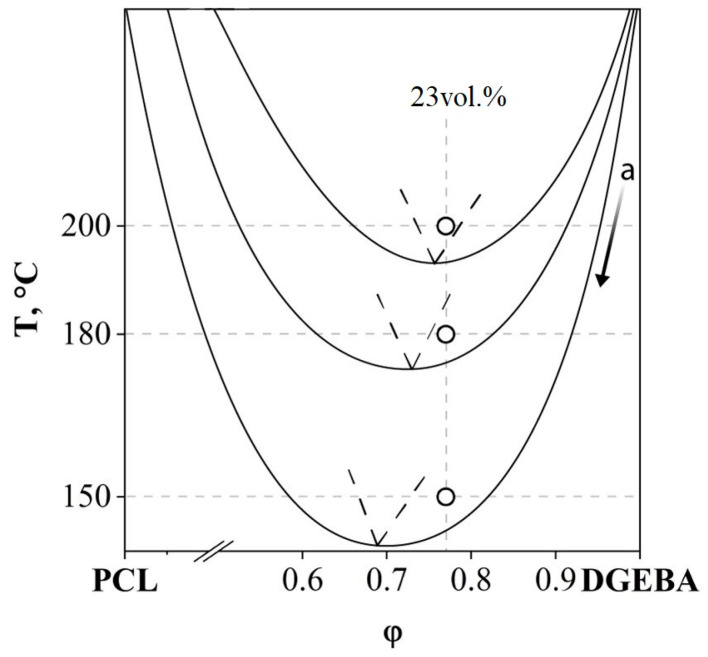
Evolution of the phase diagram of the PCL–DGEBA + DDS system during curing, illustrating the differences in the types of phase structures of systems cured at different temperatures. The dotted lines show the formation zone of the IP type.

It is important that curing a system of the same concentration at different temperatures can lead to the formation of different types of phase structures (for example, a concentration of 23 vol.%, as shown in Figure 9). This effect is associated with the evolution of the phase diagram initiated by the chemical curing reaction. According to the Flory–Huggins theory of polymer solutions, the binodal dome during the curing process expands along the concentration and temperature scales and shifts to the region of solutions enriched with a lower molecular weight component, that is, towards the thermoplastic, since the effective molecular weight of the thermoset is constantly increasing (Figure 13).

Thus, during the curing process, a figurative point with a concentration of 23 vol.%, when the dome crosses the binodal of the 200 °C isotherm, appears in the phase reversal region of the phase diagram and the formation of the IP type structure begins. Curing at a temperature of 180 °C would lead to the intersection of the isotherm with the right branch of the binodal and, as a consequence, the formation of a matrix-dispersion type structure. Curing at 150 °C is also accompanied by the formation of a matrix-dispersion structure. The difference between the structures obtained by curing the system at 150 and 180 °C lies in the ratio of the volume fractions of the forming phases. The above is confirmed by SEM images presented in Figure 9.

**Figure 14:**
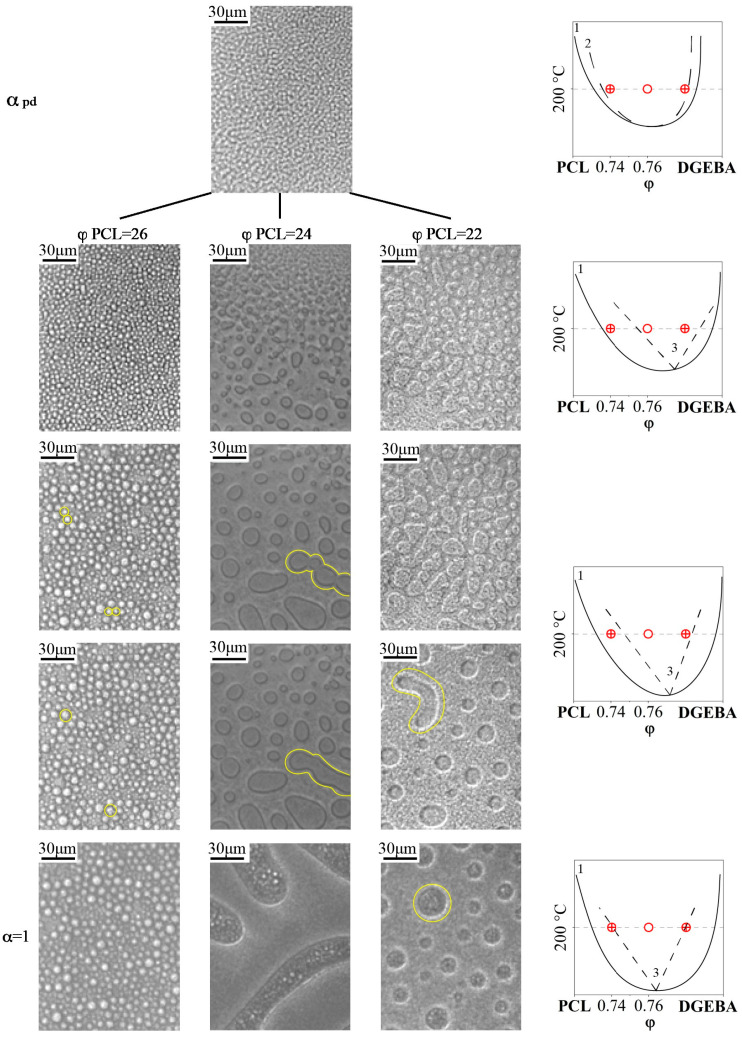
Optical microphotographs of the curing of the PCL–DGEBA + DDS mixture at T = 200 °C with PCL content of 22, 24, 26 vol.% and the corresponding model phase diagrams, where 1 is a binodal curve, 2 is a spinodal curve, 3 is a phase reversal region.

Note that the final phase structure in the cured composition is not always determined by the region of the phase diagram in which the figurative point falls when crossing the isotherm of the binodal curve. If the process of phase decomposition begins at low degrees of conversion, and, as a consequence, at high values of interdiffusion coefficients, then the displacement of the binodal dome, along the concentration scale, can lead to a change in the type of phase structure as a result of the transition of a figurative point from one region of the phase diagram to another. We studied a similar evolution of the phase structure during the curing process of the studied system in situ on an optical microscope at a temperature of 200 °C for concentrations of 22, 24 and 26 vol.%, located inside and near the phase reversal region (Figure 14).

It was found that at all studied concentrations, even with optical magnification, the phase structure at the initial stage is finely dispersed, which is in good agreement with the spinodal mechanism of phase decomposition in the region of labile solutions of the phase diagram [29,30,31]. With an increase in the degree of conversion, we observed the formation of larger phase formations from dispersed phases, followed by their merging or dissolution, depending on the movement of the binodal dome relative to the figurative points under consideration (marked with yellow lines in Figure 14). The final phase structure obtained in situ from optical microscopy studies is in good agreement with the corresponding SEM images (Figure 9). Thus, it was empirically found that in a narrow range of PCL concentrations from 23 to 25 vol.% (3 vol.%) the entire region of phase reversal of the diagram is laid out, in which the final structure of the IP type is formed.

**Figure 15:**
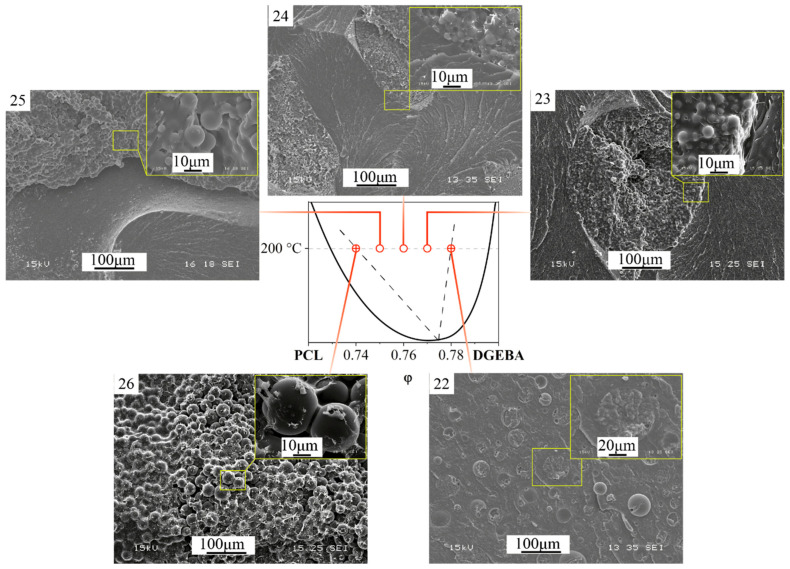
Phase structure of cured PCL–DGEBA + DDS systems at a temperature of 200 °C with PCL content: 22, 23, 24, 25 and 26 vol.% The dots indicate concentrations bordering the phase reversal region.

To clarify the morphological features during the transition to the phase reversal region, as well as to more accurately estimate its dimensions, mixtures were prepared with a PCL concentration step of 1 vol.% and cured at T = 200 °C. Figure 15 shows SEM images characteristic of the phase reversal region of the phase diagram and the concentration regions bordering it.

**Figure 16:**
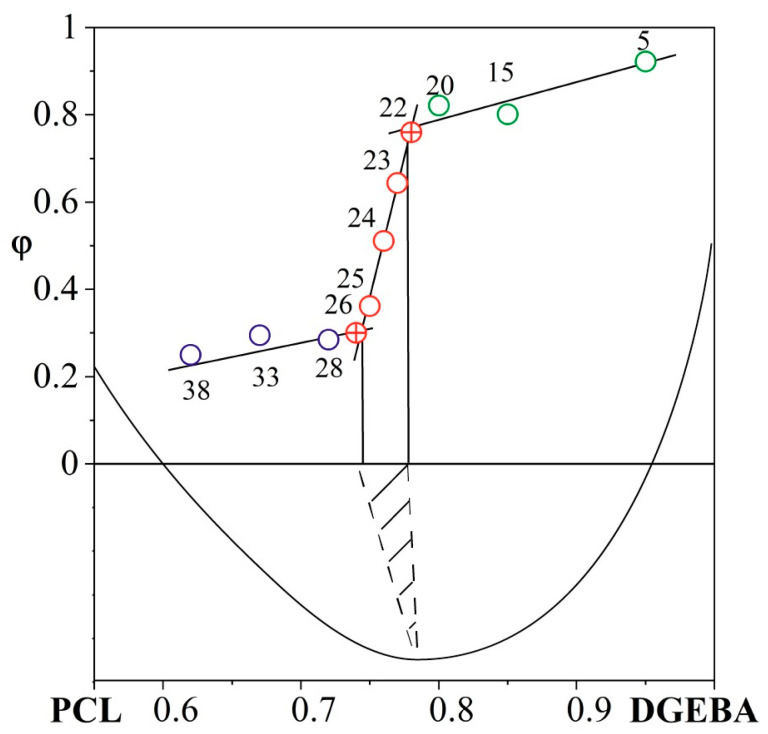
Concentration dependence of the volume fraction of DGEBA and PCL determined from SEM images of the PCL–DGEBA + DDS system cured at 200 °C. The dots indicate the volume fractions of phases of concentration systems as follows: 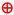 border on the phase reversal region, 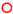 are in the phase reversal region, 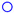 are in the inverted matrix-dispersion region, 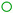 are in the matrix-dispersion region.

The volume fractions of phases enriched in DGEBA, and for the region with an inverted phase structure in PCL, calculated from SEM images were plotted against the concentration dependence (Figure 16).

Based on the intersection of the tangents, the concentrations limiting the area of formation of IP type in the system cured at 200 °C were determined. The boundary concentration values of the phase reversal region obtained from a quantitative analysis of SEM images of systems in a wide concentration range coincided with the values determined based on a qualitative analysis of microphotographs of some systems (Figure 9). Note that reducing the curing temperature from 200 to 150 °C shifts this zone to the region of thermoplastic-concentrated solutions (by ~4 vol.%) according to the data presented in Figure 9.

**Figure 17:**
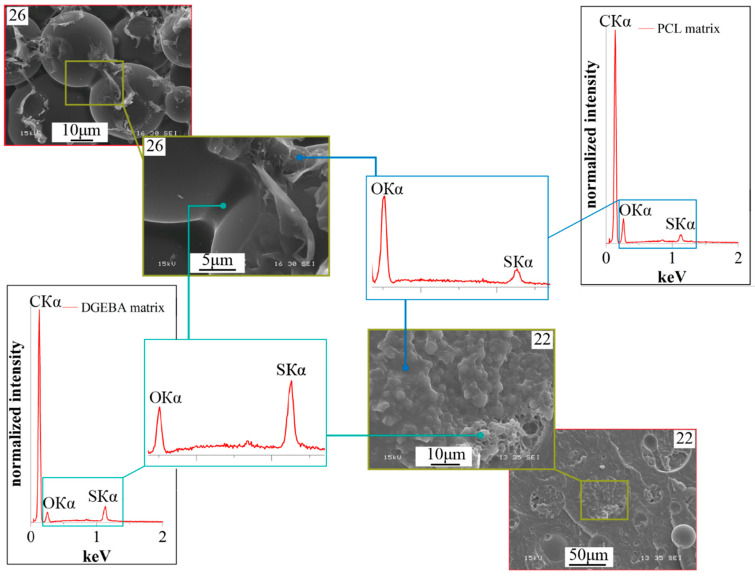
SEM image of the phase structure of the cured PCL–DGEBA + DDS system at 200 °C with PCL content of 22 and 26 vol.% and EDX spectra at points.

Also, the SEM images of the systems presented in Figure 15 made it possible to simulate the evolution of the phase structure with a smooth change in the concentration of the components. Note that, in real conditions, it is impossible to analyze changes in the phase structure with an increase/decrease in the concentration of the modifier during the chemical curing reaction. It can be seen that, in the region characterized by a matrix-dispersion type structure with inclusions enriched in PCL, when the concentration approaches the region of phase reversal (φ_PCL_ 22 vol.%), the dispersions become large and a smaller dispersed phase (“raspberry”) is identified in them according to X-ray spectral data EDX analysis enriched with epoxy oligomer (Figure 17).

**Figure 18:**
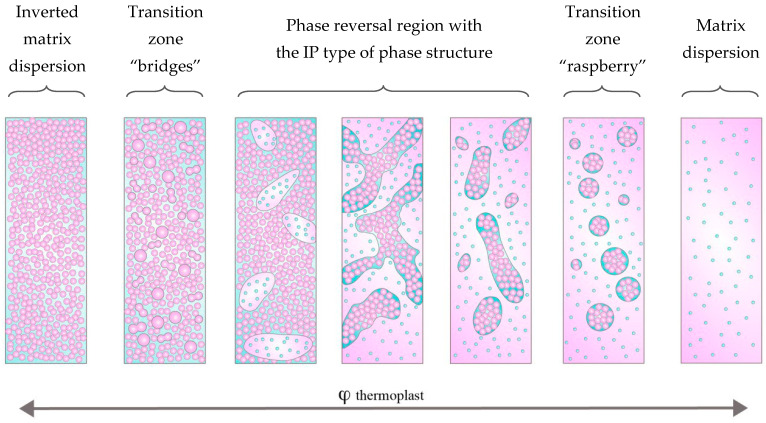
Model evolution of the phase structure with changing component concentrations.

The small phase, as shown by optical microscopy, was formed at the initial stage of phase decomposition according to the spinodal mechanism, characteristic of the phase reversal region and the zones closest to it. Further increase in PCL concentration 23, 24 and 25 vol.% leads to an increase in the size of the phase and distortion of the round cross-sectional shape, enriched in PCL and containing a finely dispersed phase enriched in epoxy resin. The volume ratio of the phases also changed towards an increase in the volume fraction of phases enriched in PCL. The evolution of the epoxy-enriched phase, which decreases in volume, looks interesting when the system concentration leaves the region of phase reversal (φ_PCL_ 26 vol.%, Figure 15). The extended epoxy phase evolves into spherical particles, which are covered with a thin layer of thermoplastic. Note that at an intermediate stage, epoxy “bridges” are observed between the spherical particles, which subsequently disappear and the structure becomes a uniform dispersion of a spherical shape enriched in epoxy, covered with a continuous matrix enriched in PCL (Figure 17). A decrease in the concentration of the thermoplastic leads to reverse processes according to the presented model of the evolution of the phase structure modified by the thermoplastic of the curing system. Figure 18 shows a diagram of the evolution of the phase structure of the cured thermoset–thermoplastic system at various thermoplastic concentrations.

### 3.3. Phase Sizes Prediction

**Figure 19:**
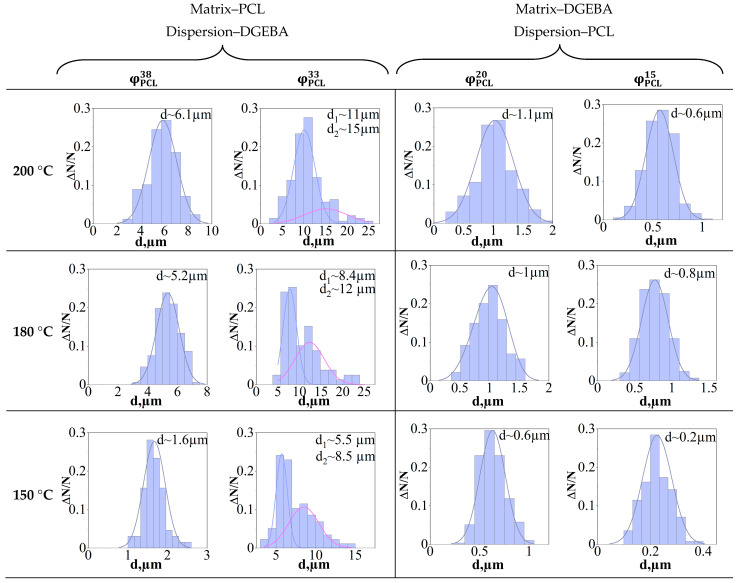
Particle size distribution (PSD) of the dispersed phase of the cured PCL–DGEBA + DDS system with PCL content: 15, 20, 33 and 38 vol.% The average particle diameter is indicated in the pictures.

An important parameter of the phase structure of a cured composition, affecting its physical and mechanical properties, is the size of the structural elements. Based on the obtained SEM images of systems with different ratios of components cured under the studied temperature conditions, correlation dependences were obtained linking the amount of modifier and the curing temperature with the sizes of the final phase structures. When analyzing the morphology, systems characterized by IP-type structures were not taken into account due to sizes differing from the sizes of phases in matrix-dispersion type structures by several tenth orders of magnitude. Images were analyzed using FIJI software (version FIJI-2.14.0) [32]. Figure 19 shows particle size distribution (PSD) histograms.

It has been established that unimodal PSD predominates in the system, and the size of dispersed phases enriched in PCL (Figure 19 φ_PCL_ 20 and 15 vol.%) and DGEBA (Figure 19 φ_PCL_ 33 and 38 vol.%) differ by 1 decimal order, which is due to macromolecular mobility components that we studied earlier in [21] on the uncured PCL–DGEBA system. The diffusion mobility of PCL in the region of solutions concentrated in epoxide is lower than the mobility of epoxide in the region of concentrations characterized by an inverted phase structure. Thus, the values of interdiffusion coefficients that determine mass transfer during phase decomposition initiated by the chemical curing reaction explain the larger size of phase particles enriched in DGEBA. Note that a decrease in the concentration of the component in the system that predominates in the dispersed phase leads to a decrease in its size. Another reason for this effect of concentration on the sizes of dispersed phases is the expansion of the heterogeneous region of the phase diagram during the curing process. Thus, the initiation of phase decomposition in systems φ_PCL_ 15 and 38 vol.% at higher degrees of conversion and, as a consequence, lower coefficients of interdiffusion and complicated mass transfer when the system equalizes the composition of coexisting phases to equilibrium.

The fact of a smaller change in the size of the dispersed phase enriched with thermoplastic follows from the position of the binodal curve of the phase diagram of the system during the curing process. The binodal dome is asymmetrical and shifted towards solutions concentrated according to DGEBA in full accordance with the Flory–Huggins theory of polymer solutions. In this regard, with an increase in the degree of conversion, the expansion of the heterogeneous region is realized to a greater extent due to the shift of the left branch of the binodal, which determines the composition of the dispersed phase enriched in DGEBA.

An important feature of the structure formation of systems characterized by an inverted phase structure of the matrix-dispersion type with inclusions enriched in DGEBA is the formation of a bimodal distribution of phase structures. This feature of structure formation appears in systems close in concentration to systems with a structure of the IP type. This is due to the onset of phase decomposition at low conversion rates and, as a consequence, high diffusion coefficients, which promote the formation of larger phase particles, along with smaller ones in the later stages of curing. Also, small phase structures enriched in DGEBA are formed in systems with a higher concentration of thermoplastic, which is a consequence of the expansion of the heterogeneous region of the phase diagram during curing and the initiation of phase decomposition at later stages of the chemical reaction.

When considering the effect of curing temperature on the dimensions of the formed phase structures, it is also important to take into account the degree of conversion of the onset of phase decomposition and the rate of the chemical reaction of curing, which is a function of temperature. Since the PCL–DGEBA + DDS system is characterized by an amorphous–crystalline diagram with LCST, the intersection of the 150 °C curing isotherm occurs at a later stage of the curing reaction and, as a result, lower diffusion coefficients than the 200 °C isotherm. This is in good agreement with the PSD data, where a decrease in the curing temperature is accompanied by a decrease in the size of phase particles.

**Figure 20:**
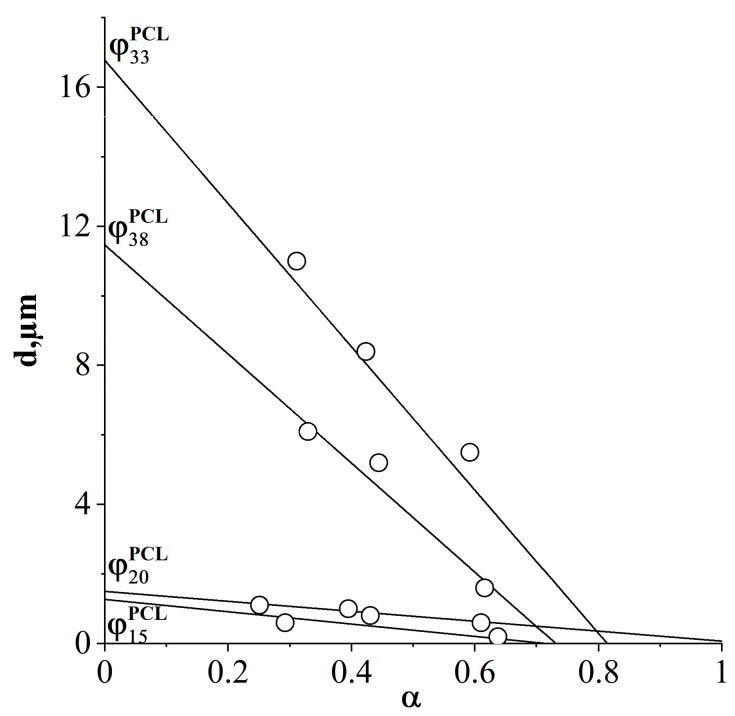
Dependence of the size of the formed dispersed phases on the degree of conversion of the beginning of phase decomposition. PCL content: 15, 20, 33, 38 vol.%.

Based on the obtained average diameters of dispersed phase particles for systems with different ratios of components, cured at 150, 180 and 200 °C (Figure 19), and the dependence from Figure 8a, the influence of the degree of conversion of the onset of phase decomposition on the size of dispersed phase structures in cured compositions at various concentrations of components in a homogeneous solution was determined (Figure 20).

The obtained dependence allows us to predict the sizes of phase structures for systems characterized by a matrix-dispersion type structure. Summarizing the data, we can predict that systems with a PCL concentration of no more than 20 vol.% thermoplastic-enriched dispersed particles with sizes of no more than 2 microns are formed. Reducing the curing temperature and thermoplastic concentration leads to a decrease in the size of the phase particles. When moving to systems with an inverted phase structure corresponding to PCL concentrations above 26 vol.%, large phases are formed, the size of which, in addition to the PCL concentration, is affected by the degree of conversion of the onset of phase decomposition, that is, the distance between the critical temperature of the uncured system and the curing temperature. A decrease in the curing temperature and, as a consequence, an increase in the degree of conversion of the onset of phase decomposition leads to a decrease in the size of dispersed phase structures.

### 3.4. The Composition of Coexisting Phases Prediction

Above, we defined qualitative parameters and their quantitative laws that affect the type of phase structure and the sizes of structural elements formed during the curing of the thermoplastic system. As has already been shown above, during the curing process, the binodal curve of the phase diagram expands along the concentration and temperature scales, and the interdiffusion coefficients decrease. And since the formation of phases when the figurative point of the system enters the heterogeneous region of the phase diagram is influenced by macromolecular mobility, the degree of conversion of the completion of the chemical reaction in the modified system and the composition of the coexisting phases fixed in the solidified structure is of fundamental importance.

**Figure 21:**
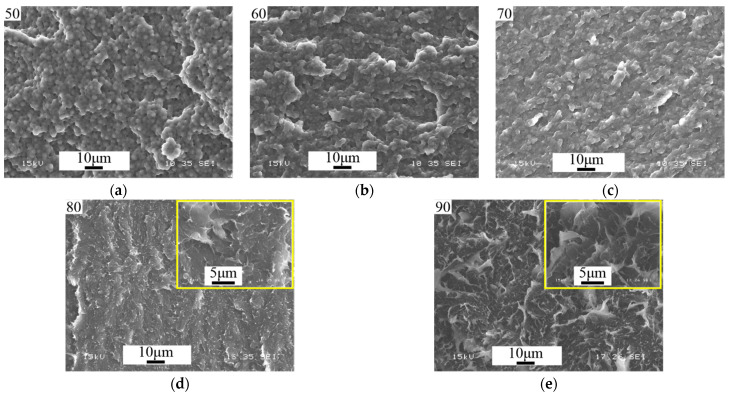
SEM images of the phase structure of cured PCL–DGEBA + DDS systems at 200 °C. The PCL contents ( vol.%) in the mixtures are indicated in the pictures: 50 (**a**), 60 (**b**), 70 (**c**), 80 (**d**) and 90 (**e**).

For this purpose, PCL–DGEBA + DDS mixtures with a PCL content of 50–90 vol.% in step of 10 vol.% were studied. SEM images of cured systems are presented in Figure 21.

**Figure 22:**
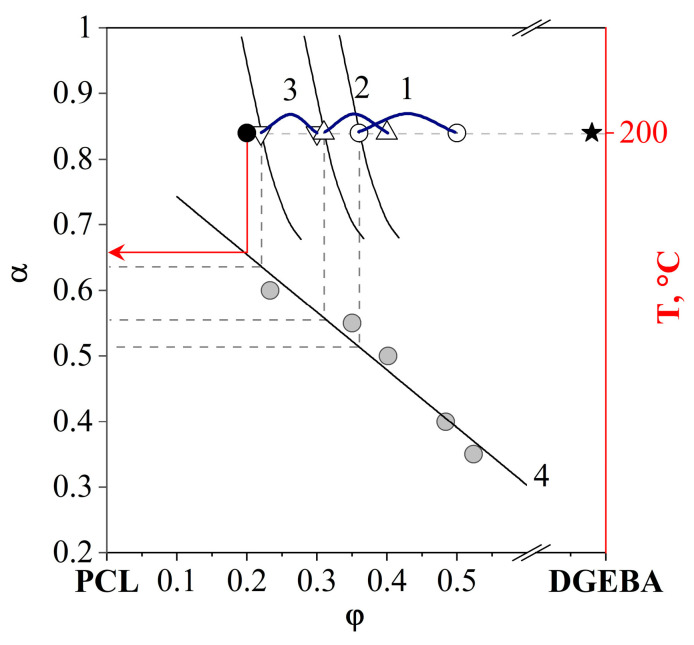
Generalized data on the composition of the phase enriched with PCL and the degree of conversion of cured systems depending on the concentration of the initial components in the PCL–DGEBA + DDS system 50 (○), 60 (△), 70 (▽) and 80 vol.% (●). ★ corresponds to a fixed composition of the phase enriched with DGEBA 2 vol.% 1, 2, 3—canodes corresponding to the volume fraction of the phase enriched in DGEBA. 4—concentration dependence on the degree of conversion of the cured system.

Analysis of SEM images of thermoplastic-concentrated cured solutions showed that there are no regular phase particles in systems containing 90 and 80 vol.% PCL. It is important that the system with 50 vol.% PCL, on the contrary, is characterized by a heterogeneous phase structure of the matrix-dispersion type with a volume fraction of the dispersed phase of 0.24. This information allows us to determine with good accuracy, using the “lever” rule, the composition of the matrix, which is characterized by the left branch of the binodal curve [33]. The accuracy of determining the composition is due to the asymmetry of the binodal curve (φ^cr^_DGEBA_ = 0.751 at 200 °C) and, as a consequence, the right branch of the binodal curve being pressed to the temperature axis of the diagram. We applied this algorithm for calculating the composition of coexisting phases to other heterogeneous systems with an inverted matrix-dispersion phase structure. Thus, the PCL–DGEBA + DDS system with PCL concentrations of 50, 60 and 70 vol.% is characterized by a dispersed phase composition close to pure DGEBA and a matrix composition of 64, 69 and 78 vol.% PCL, respectively. Note that the position of the left branch of the binodal curve did not reach the system with a PCL concentration of 80 vol.%, which confirms the SEM data on the homogeneity of systems with a PCL content of 80 vol.% or more (Figure 21d,e). The concentration dependence of the degree of conversion corresponding to the phase structure cured at 200 °C was determined above from interferometry data (Figure 8b). Generalized data on the composition and degree of conversion of cured matrices depending on the concentration of the initial components in the PCL–DGEBA + DDS system are presented in Figure 22 and Table 3.

It can be seen that the higher the concentration of thermoplastic in the system, the higher the degree of conversion of epoxy groups in the matrix of cured systems enriched with thermoplastic. This is explained by the preservation of diffusion mobility at high degrees of conversion due to the plasticization of the system with thermoplastic. As noted above, in systems with 80 vol.% PCL phase decomposition is not observed. And, according to the scheme (Figure 22), diffusion difficulties occur at a degree of conversion of epoxy groups of 0.65.

Thus, we determined the concentration of PCL in the system, above which the mixtures will remain homogeneous after curing. Cured systems with inverted phase structure and thermoplastic concentration below 80 vol.% are characterized by a heterogeneous structure. From the abovementioned, it follows that, depending on the concentration of the initial components, the final phase structure is characterized by both different compositions of the coexisting phases and different cross-linking densities of the systems, which will affect the physical and mechanical properties of the system.

**Figure 23:**
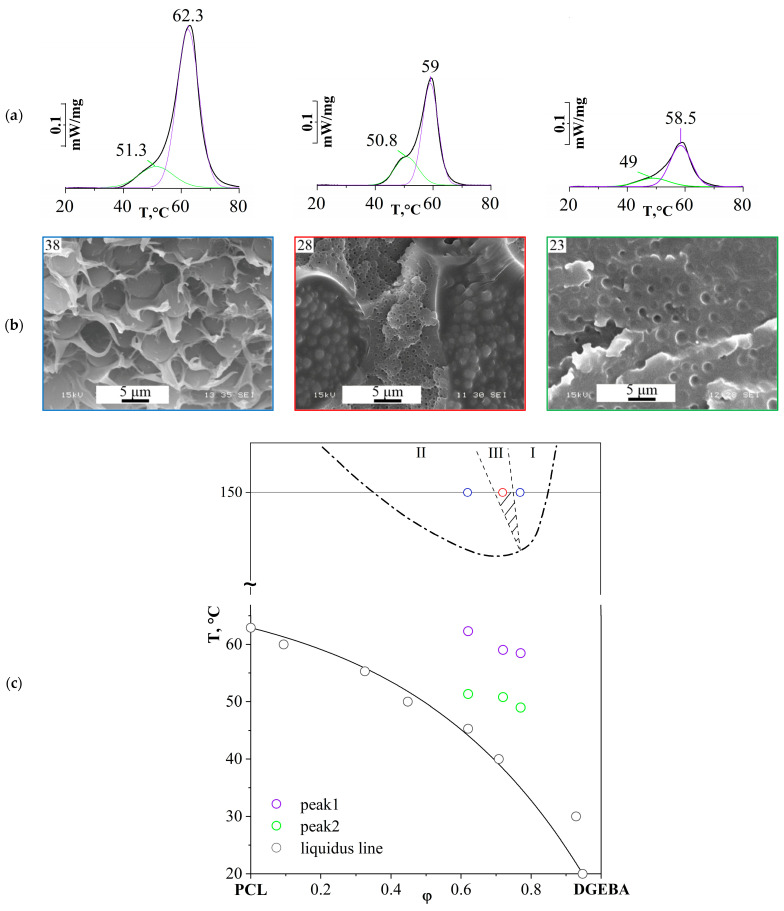
(**a**) DSC thermograms of PCL–DGEBA + DDS mixtures cured at 150 °C with PCL content: 23, 28, 38 vol.%; (**b**) morphology of the PCL–DGEBA + DDS mixture with PCL content: 23, 28, 38 vol.%; (**c**) generalized phase diagram of the PCL–DGEBA + DDS system. Regions: I—dispersion matrix, III—phase reversal region, II—phase inversion. The dots mark the melting temperatures of cured PCL–DGEBA + DDS mixtures containing PCL of 23, 28, 38 vol.%, as well as the liquidus line corresponding to the melt solutions of the PCL–DGEBA mixture [21].

Additionally, the phase nature of the coexisting phases was assessed for the content of crystallizing structures in them. For this purpose, thermograms were obtained for various compositions of the cured PCL–DGEBA + DDS system, with different types of phase structures: the matrix is enriched with DGEBA with inclusions enriched with PCL and the matrix is enriched with PCL with inclusions—DGEBA, coexisting interpenetrating (continuous) matrices—enriched with PCL and DGEBA DSC (Figure 23).

The greatest difference in the values characterizing phase transitions can be achieved at a curing temperature of 150 °C, that is, when the formation of phase structures begins at a higher degree of conversion and a lower rate of chemical reaction compared to the temperatures of 180 and 200 °C. It was found that all systems are characterized by two melting peaks, which indicates the presence of crystalline structures in both coexisting phases of the systems under study (Figure 23a). The values of the maximum melting temperatures (T_m_), as well as the liquidus line of the initial uncured system [21], are presented in the temperature–concentration field (Figure 23c).

The obtained melting point values characterize the quantitative content of crystallizing thermoplastic in the phase and are given in Table 4.

Note that for an inverted phase structure of the matrix-dispersion type (PCL content 38 vol.%), T_m_ = 62.6 °C is close to the peak melting temperature of pure PCL (62.9 °C) since the matrix is predominantly PCL. The presence of the second peak indicates that the dispersed spherical phase, enriched in thermoset, also contains a thermoplastic component. In the case of the obtained dependencies for PCL 28 vol.% with the IP-type phase structure, a more pronounced left shoulder is observed in the thermogram, which is due to the appearance of a dispersed phase enriched in PCL in one of the continuous phases enriched in DGEBA.

Thus, the phase nature of coexisting phases in cured systems, regardless of the type of phase organization, can be defined as amorphous–crystalline. Note that phases enriched with thermoplastic have a higher melting point, and with increasing concentration of the epoxy oligomer in the initial mixture, a depression in the melting temperatures in both coexisting phases is observed.

## 4. Conclusions

In this study, we showed the possibility of regulating the phase structure, phase composition and structural and morphological parameters of the final material at the stage of determining the concentration of components and temperatures of the technological process of curing a reactive thermoplastic system.

The solution to the problem of regulating the phase structure and its parameters in bicomponent curing mixtures was carried out through a comprehensive study of the model curing by DDS amorphous–crystallizing system PCL–DGEBA using electron and optical microscopy, optical interferometry, FTIR spectroscopy and DSC.

It is shown that the mixing of the initial components of the PCL–DGEBA system is described by the crystal equilibrium phase diagram. Solidification of the components leads to the appearance of amorphous separation with LCST, and the system is described by a mixed amorphous–crystalline equilibrium.

A method for determining the critical concentration that determines the middle of the phase reversal region on the phase diagram has been developed and tested. It has been shown that cured mixtures of this composition will be characterized by a structure of the type of interpenetrating phase. It has been established that, with good mobility of the system, if the initiation of phase decomposition occurs at low degrees of conversion of epoxy groups, the final structure may differ from the predicted one due to a shift in the critical concentration of the phase diagram towards the thermoplastic. The influence of curing temperature on the critical concentration was determined. For the studied system, a change in the curing temperature by 50 °C leads to a shift in the critical concentration by 4 vol.% The dependences of critical concentrations and the degree of conversion of the onset of phase decomposition on the curing temperature were obtained. Concentration ranges for mixing components corresponding to solidified structures such as interpenetrating phases, matrix dispersion and inverted matrix dispersion have been determined.

The proposed approaches have been tested by studying the phase structure in a wide temperature and concentration range. The structure formation in the phase reversal region of the phase diagram was studied and its width was determined, the component for the PCL–DGEBA + DDS system cured at 200 °C is 3 vol.% from 22 to 26 vol.%, where systems with modifier concentrations of 22 and 26 vol.% are borderline. The structure of systems bordering the phase reversal region is designated by the authors as “raspberries” when transitioning from the matrix-dispersion structure, and as “bridges” when transitioning from the inverted matrix-dispersion structure.

Histograms of particle size distribution were constructed and generalized into correlation dependences of the sizes of dispersed structures on the degree of conversion of the onset of phase decomposition for different ratios of the initial components in the PCL–DGEBA mixture. It has been shown that the final size of dispersed particles enriched with both thermoplastic and thermoset is influenced by the distance between the critical temperature of the phase diagram of the initial components and the curing temperature. The difference in the sizes of dispersed structures in the matrix-dispersion and inverted matrix-dispersion systems is determined by the diffusion mobility of PCL in the DGEBA matrix and DGEBA in the PCL matrix, respectively. Thus, the size of the dispersed phase enriched in DGEBA can exceed 10 µm, and the phase enriched in PCL does not exceed 2 µm.

The composition of coexisting phases was studied based on the analysis of SEM images and DSC thermograms. The concentration of PCL in the system was determined, above which the mixtures will remain homogeneous after curing. It is shown that, depending on the concentration of the initial components, the final phase structure will be characterized by both different compositions of the coexisting phases and different cross-linking densities of the systems, which will affect the physical and mechanical properties of the system.

The approaches used in this study are universal in nature and can be applied to create materials with a given type of phase organization, the sizes of structural elements and the compositions of coexisting phases based on curing thermoset–thermoplastic systems. Targeted regulation of the phase structure of multicomponent polymer systems will make it possible to develop binders of polymer composite materials that have the required set of performance characteristics, bypassing routine screening studies.

## Figures and Tables

**Table 1:** Systems, conditions and methods of their research.

System	φ_PCL_, vol.%	T, °C	Method					
			OI	DSC	FTIR	SEM	OM	EDX
Study of the system during the curing process								
PCL with DGEBA + DDS	–	150, 180, 200	+					
PCL with aDGEBA (α: 0.3–0.6)	–	140–200	+					
PCL–DGEBA + DDS	28, 26, 24	10 °C/min		+				
	22, 24, 26	200					+	
Study of the cured system								
DGEBA + DDS	0	200			+			
PCL–DGEBA + DDS	26	180			+			
	23, 28, 38 (10 °C/min)	150	+					
	15, 20, 23, 28, 33, 38	150				+		
	15, 20, 23, 26, 33, 38	180				+		
	15, 20, 22, 23, 24, 25, 26, 28, 33, 38, 50, 60, 70, 80, 90	200				+		
	22, 24, 26	200						+

**Table 2:** Time of onset of phase decomposition and degree of conversion of the curing mixture PCL–DGEBA + DDS.

T_cur_, °C	φ_PCL_, vol.%	Changing the Inclination Angle		Phase Decomposition
		t_gel_, min (according to OI Data)	α_gel_, (according to DSC)	t_pd_, min (according to OI Data)
150	28	26	0.21	87
180	26	11	0.31	16
200	24	5	0.30	9

**Table 3:** Generalized data on the composition of the enriched PCL phase and the degree of conversion of cured PCL–DGEBA + DDS systems depending on the concentration of the initial components.

PCL–DGEBA + DDS φ_PCL_, vol.%	α	ϕ_DGEBA_	φ_PCL_, vol.%	α
	The Beginning of Phases Formation (from Line 4 in Figure 18)	From SEM Images	Calculation according to the “Lever” Rule	Fixation of Structure (from Line 4 in Figure 23)
50 (○)	0.39	0.238	64 (1)	0.52
60 (△)	0.48	0.130	69 (2)	0.56
70 (▽)	0.56	0.118	78 (3)	0.63
80 (●)	0.65	0	-	-

**Table 4:** Melting point and enthalpy values for the PCL–DGEBA + DDS mixture cured at 150 °C.

	$\varphi_{PCL}^{38}$ (∆H = 26.68 J/g)		$\varphi_{PCL}^{28}$ (∆H = 14.18 J/g)		$\varphi_{PCL}^{23}$ (∆H = 7.35 J/g)	
	peak1	peak2	peak1	peak2	peak1	peak2
**T_m_, °C**	62.3	51.3	59	50.8	58.5	49
**∆H, J/g**	21.32	5.36	7.92	6.26	5.36	1.99

## Data Availability

Data are contained within the article and Supplementary Materials.

## Supplementary Materials

The following supporting information can be downloaded at: https://www.mdpi.com/article/10.3390/polym16192695/s1, Figure S1: Original version of Figure 9 in main text: (A) T_cur_ = 200 °C, 38 vol.%; (B) T_cur_ = 200 °C, 33 vol.%; (C) T_cur_ = 200 °C, 24 vol.%; (D) T_cur_ = 200 °C, 23 vol.%; (E) T_cur_ = 180 °C, 38 vol.%; (F) T_cur_ = 180 °C, 33 vol.%; (G) T_cur_ = 180 °C, 26 vol.%; (H) T_cur_ = 180 °C, 23 vol.%; (I) T_cur_ = 150 °C, 38 vol.%; (J) T_cur_ = 150 °C, 33 vol.%; (K) T_cur_ = 150 °C, 28 vol.%; (L) T_cur_ = 150 °C, 23 vol.%.; Figure S2: Original version of Figure 12 in main text: (A) T_cur_ = 200 °C, 24 vol.%; (B) T_cur_ = 200 °C, 24 vol.%; (C) T_cur_ = 200 °C, 24 vol.%.; Figure S3: Original version of Figure 15 in main text: (A) T_cur_ = 200 °C, 22 vol.%; (B) T_cur_ = 200 °C, 22 vol.%; (C) T_cur_ = 200 °C, 23 vol.%; (D) T_cur_ = 200 °C, 23 vol.%; (E) T_cur_ = 200 °C, 24 vol.%; (F) T_cur_ = 200 °C, 24 vol.%; (G) T_cur_ = 200 °C, 25 vol.%; (H) T_cur_ = 200 °C, 25 vol.%; (I) T_cur_ = 200 °C, 26 vol.%; (J) T_cur_ = 200 °C, 26 vol.%.; Figure S4: Original version of Figure 17 in main text: (A) T_cur_ = 200 °C, 26 vol.%; (B) T_cur_ = 200 °C, 26 vol.%; (C) T_cur_ = 200 °C, 22 vol.%; (D) T_cur_ = 200 °C, 22 vol.%.; Figure S5: Original version of Figure 21 in main text: (A) T_cur_ = 200 °C, 50 vol.%; (B) T_cur_ = 200 °C, 60 vol.%; (C) T_cur_ = 200 °C, 70 vol.%; (D) T_cur_ = 200 °C, 80 vol.%; (E) T_cur_ = 200 °C, 80 vol.%; (F) T_cur_ = 200 °C, 90 vol.%; (G) T_cur_ = 200 °C, 90 vol.%.; Figure S6: Original version of Figure 23 in main text: (A) T_cur_ = 150 °C, 38 vol.%; (B) T_cur_ = 150 °C, 28 vol.%; (C) T_cur_ = 150 °C, 23 vol.%.
