# Supplementary material for: Regulation of the Phase Structure in the Crystallizing Curing System PCL–DGEBA
Source: Polymers (Basel). 2024 Sep 24;16(19):2695. doi: 10.3390/polym16192695 (PMC11478844; doi:10.3390/polym16192695)
Supplement: Supplementary file 1 [file polymers-16-02695-s001.zip › polymers-3201256-supplementary.pdf]

Supplementary materials (raw SEM photos) for article "Regulation of the Phase Structure in the Crystallizing Curing System PCL–DGEBA" by Irina O. Plyusnina, Uliana V. Nikulova, Ramil R. Khasbiullin and Aleksey V. Shapagin.

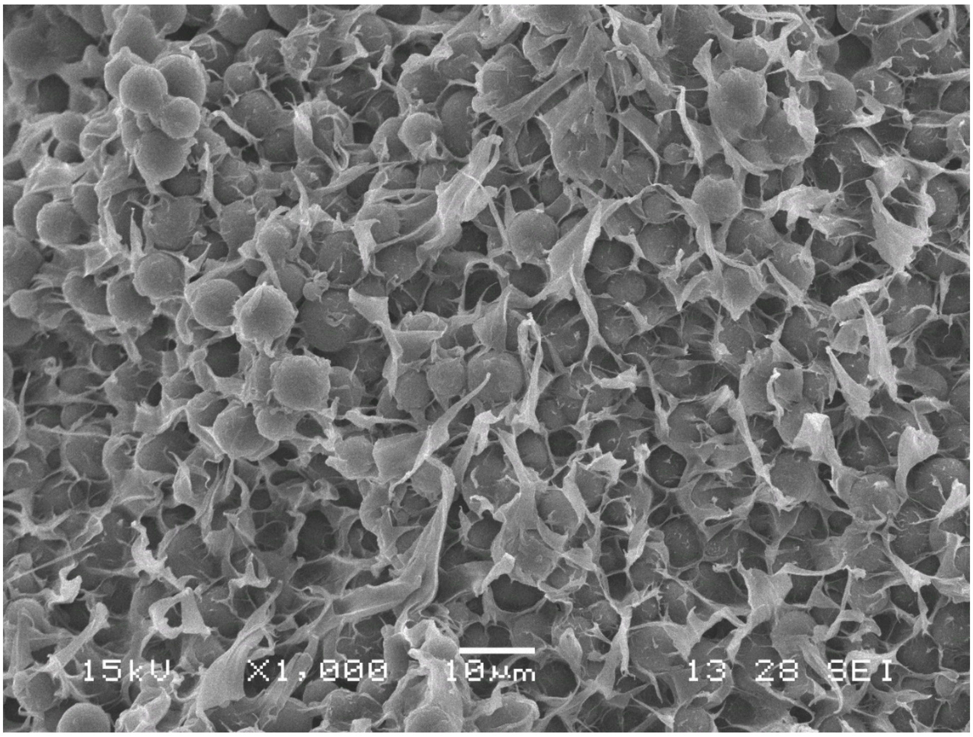

(A)

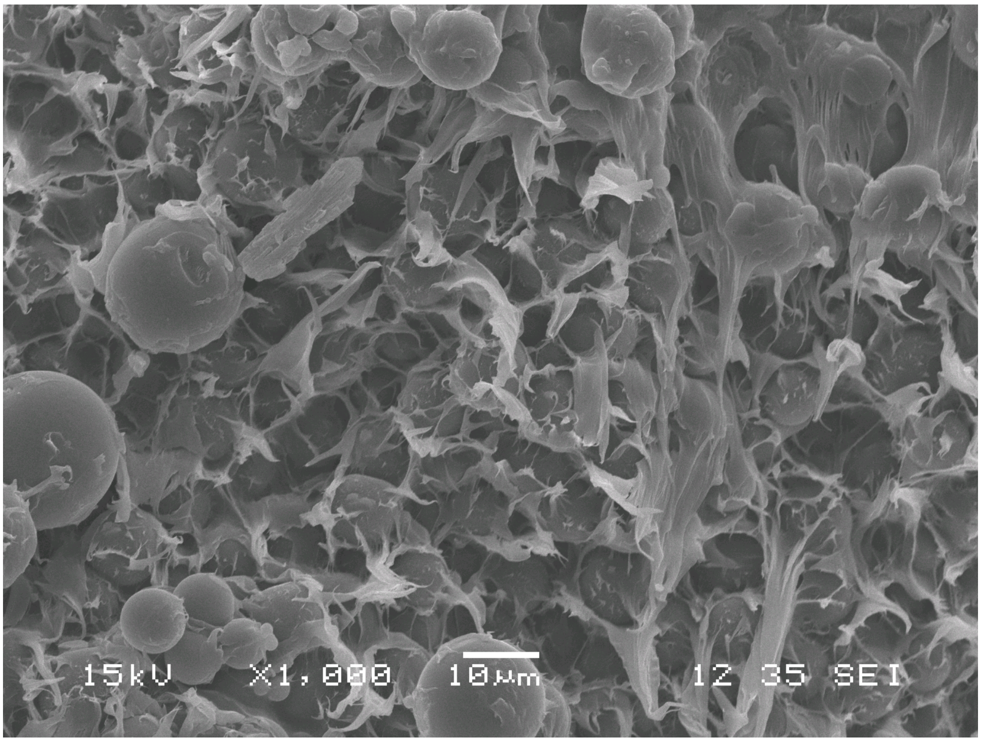

(B)

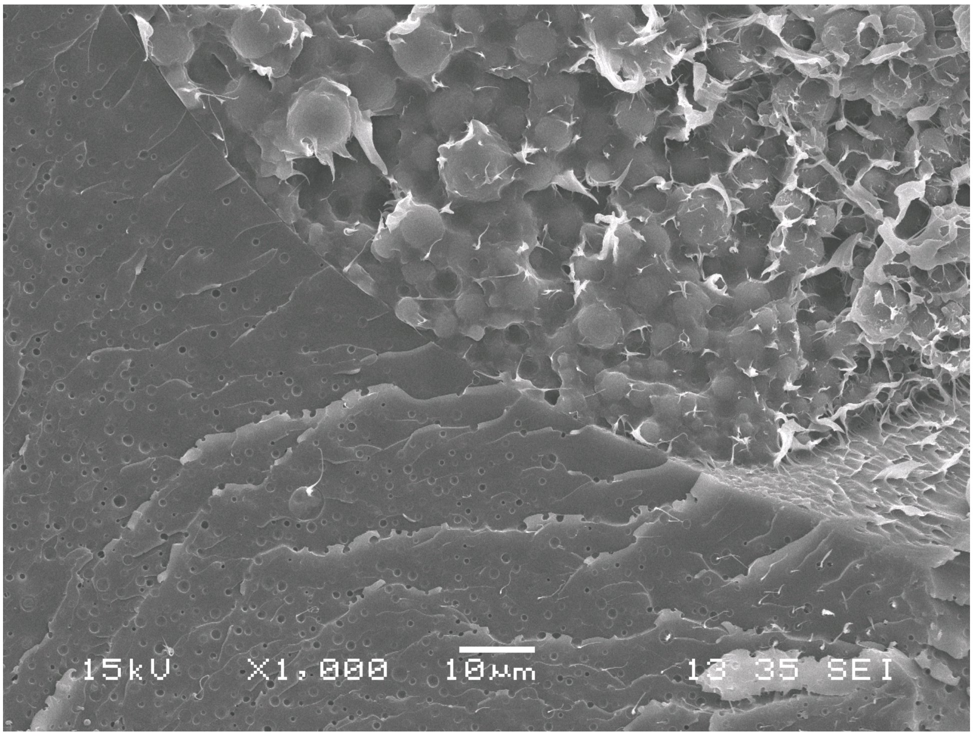

(C)

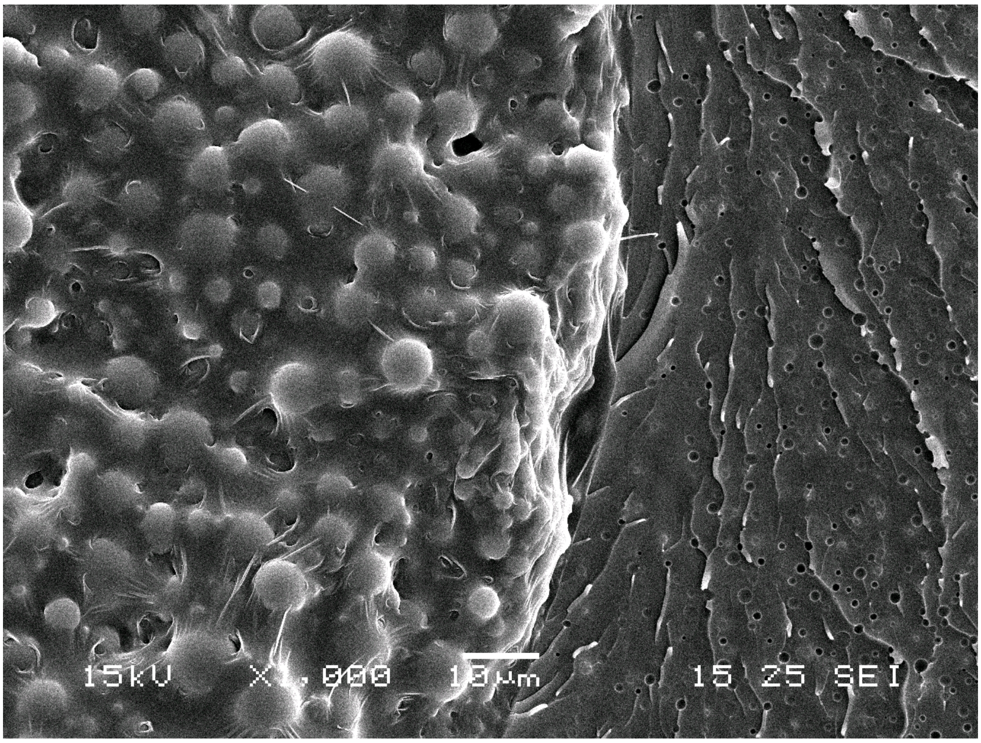

(D)

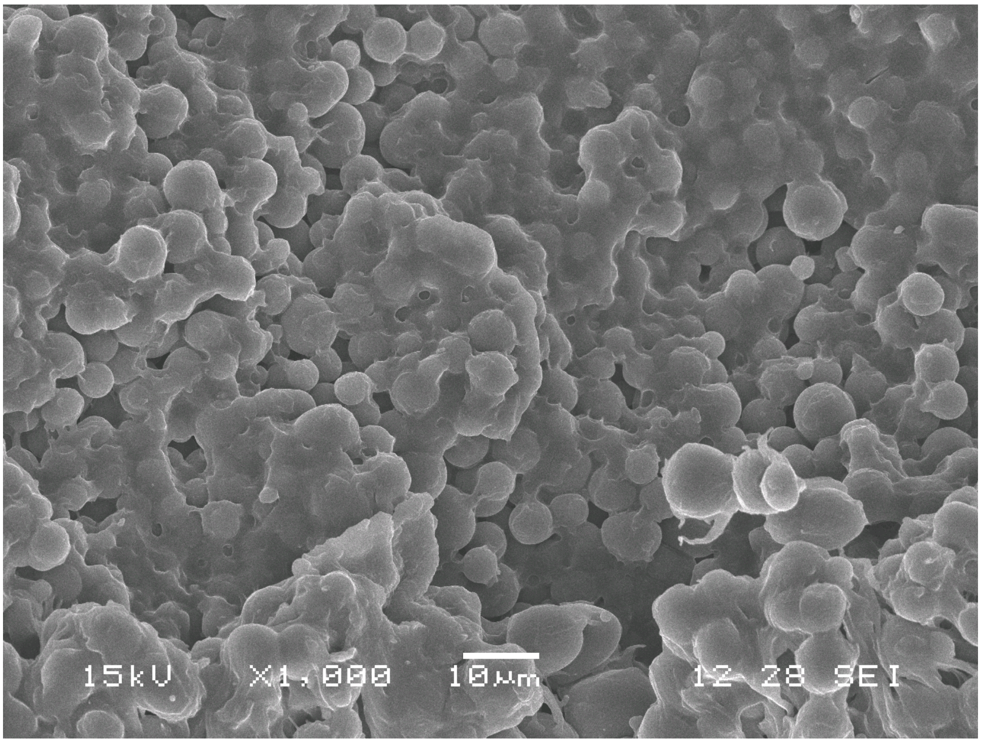

(E)

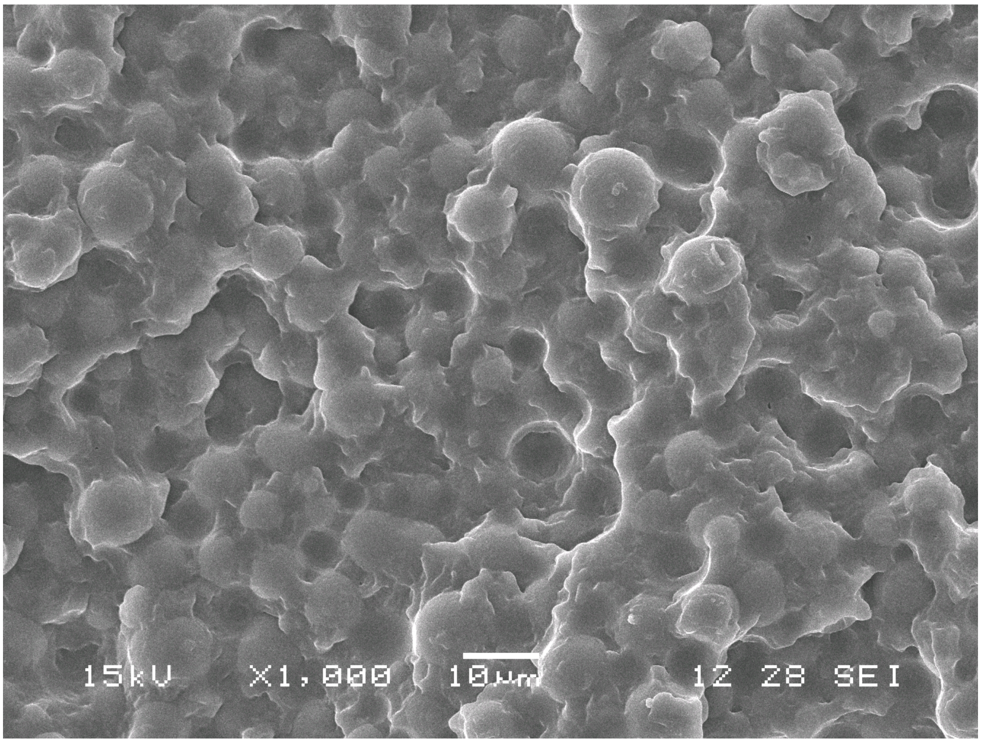

(F)

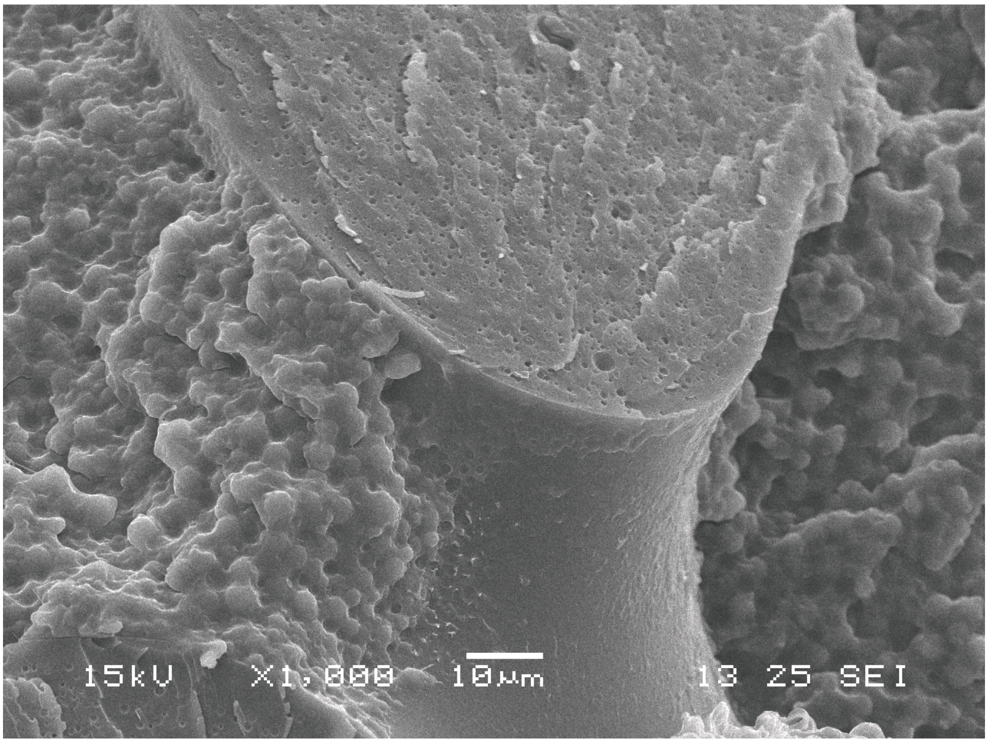

(G)

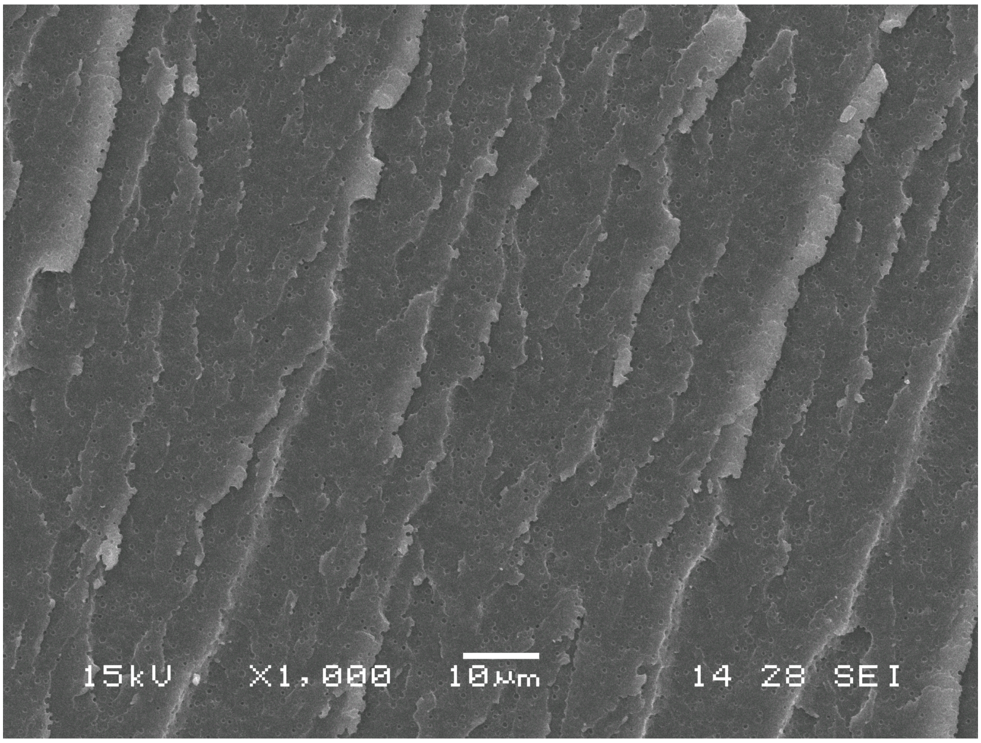

(H)

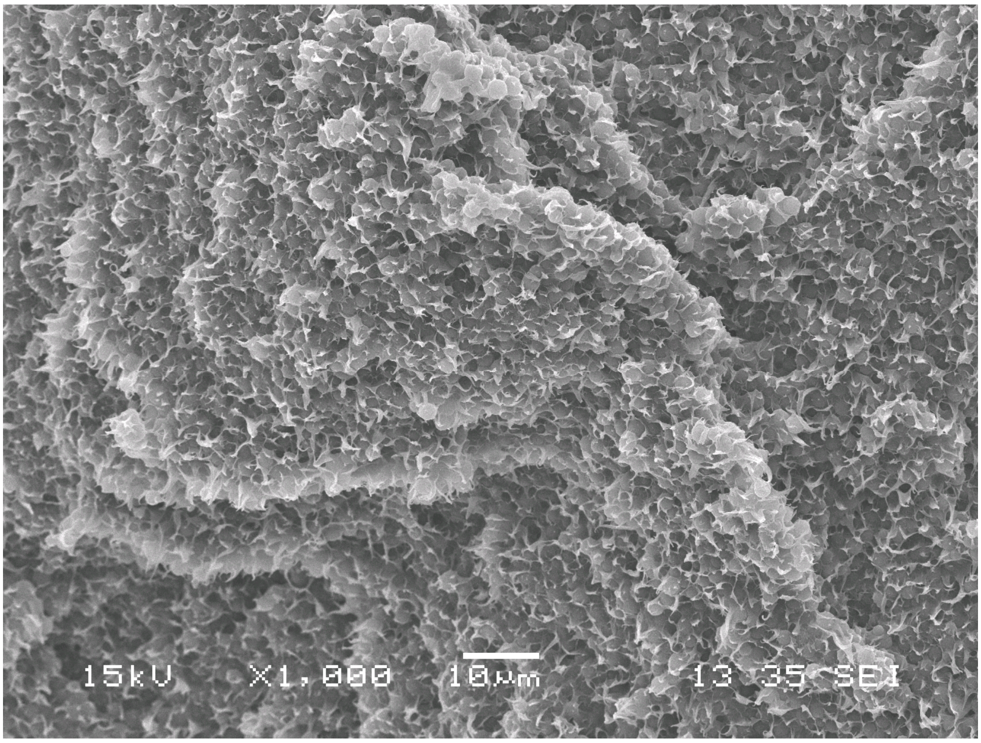

(I)

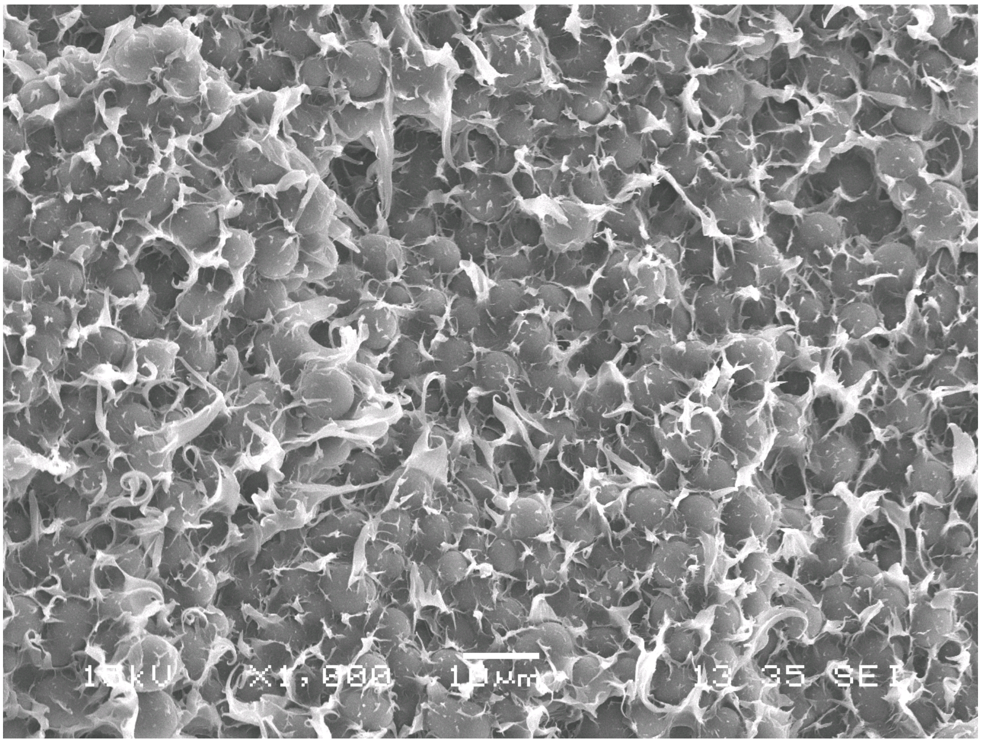

(J)

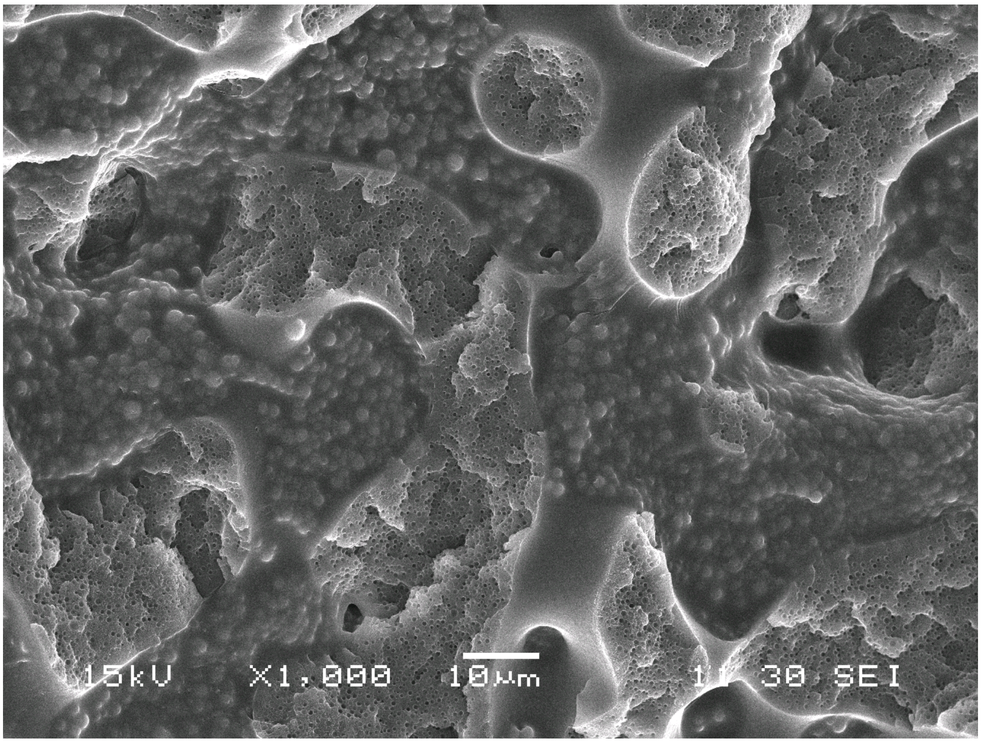

(K)

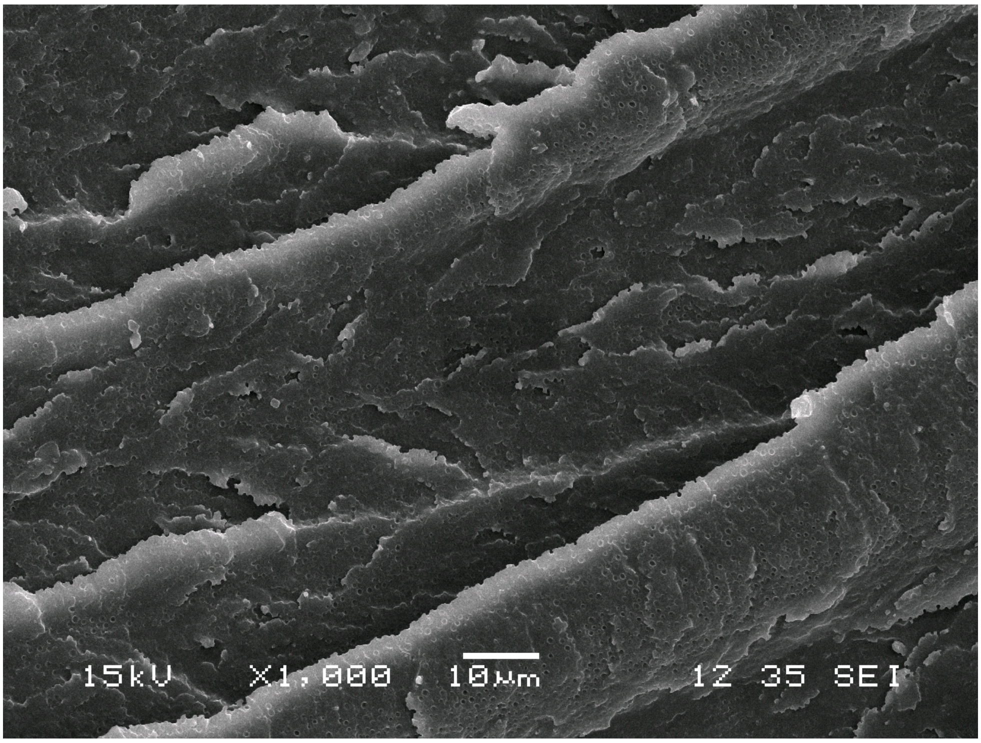

(L)

Figure S1. Original version of Figure 9 in main text: (A) T_{cur} = 200 °C, 38vol.%; (B) T_{cur} = 200 °C, 33vol.%; (C) T_{cur} = 200 °C, 24vol.%; (D) T_{cur} = 200 °C, 23vol.%; (E) T_{cur} = 180 °C, 38vol.%; (F) T_{cur} = 180 °C, 33vol.%; (G) T_{cur} = 180 °C, 26vol.%; (H) T_{cur} = 180 °C, 23vol.%; (I) T_{cur} = 150 °C, 38vol.%; (J) T_{cur} = 150 °C, 33vol.%; (K) T_{cur} = 150 °C, 28vol.%; (L) T_{cur} = 150 °C, 23vol.%.

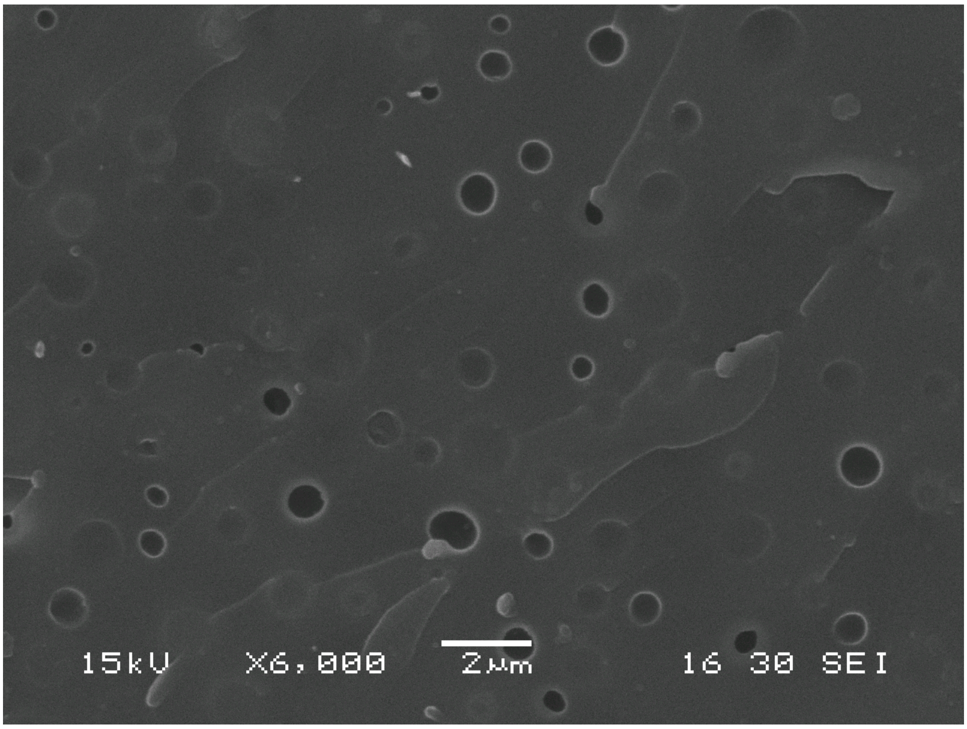

(A)

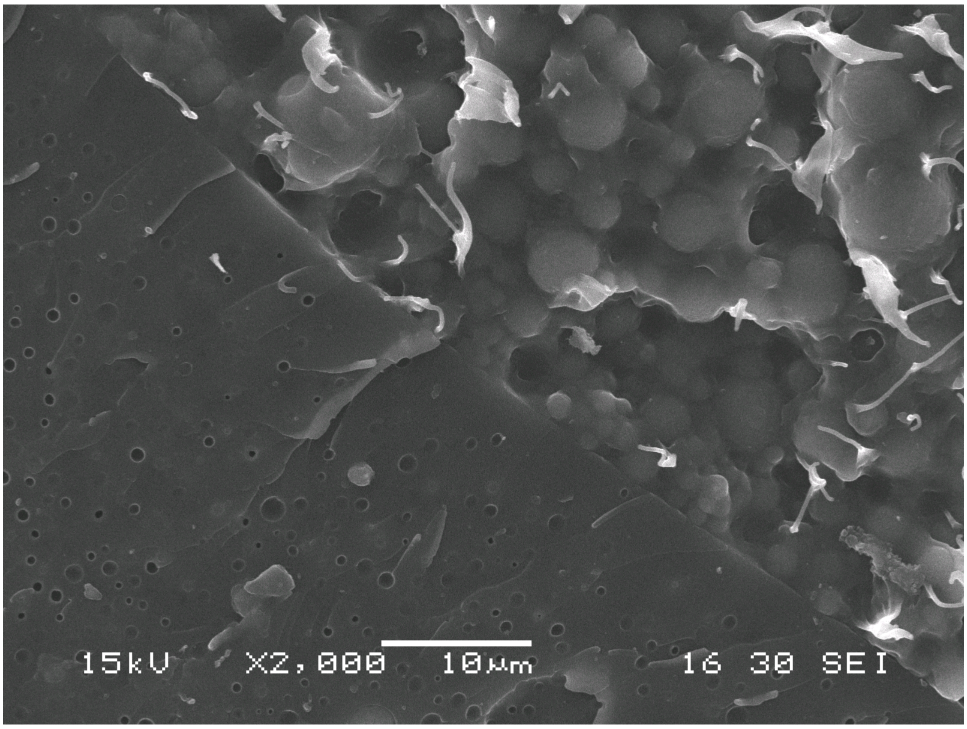

(B)

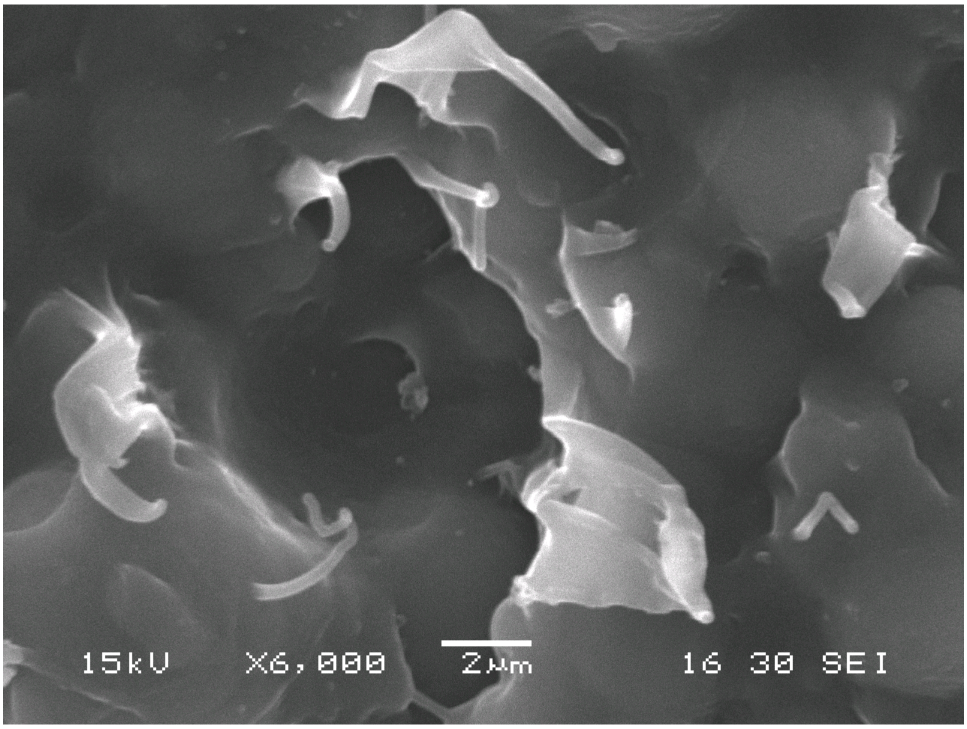

(C)

Figure S2. Original version of Figure 12 in main text: (A) T_{cur} = 200 °C, 24vol.%; (B) T_{cur} = 200 °C, 24vol.%; (C) T_{cur} = 200 °C, 24vol.%.

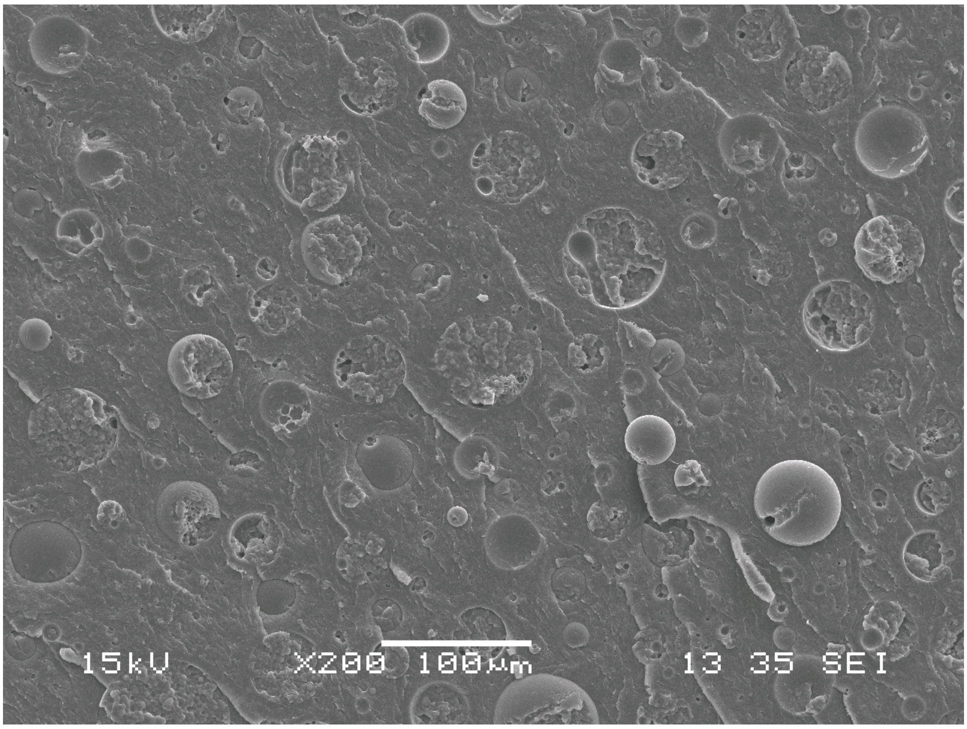

(A)

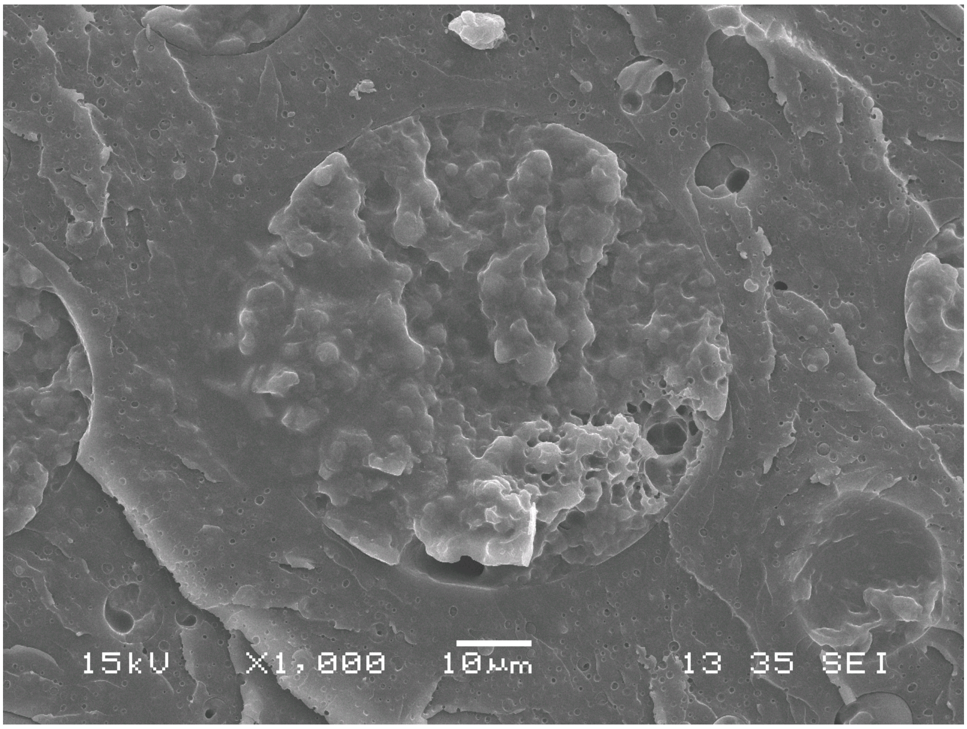

(B)

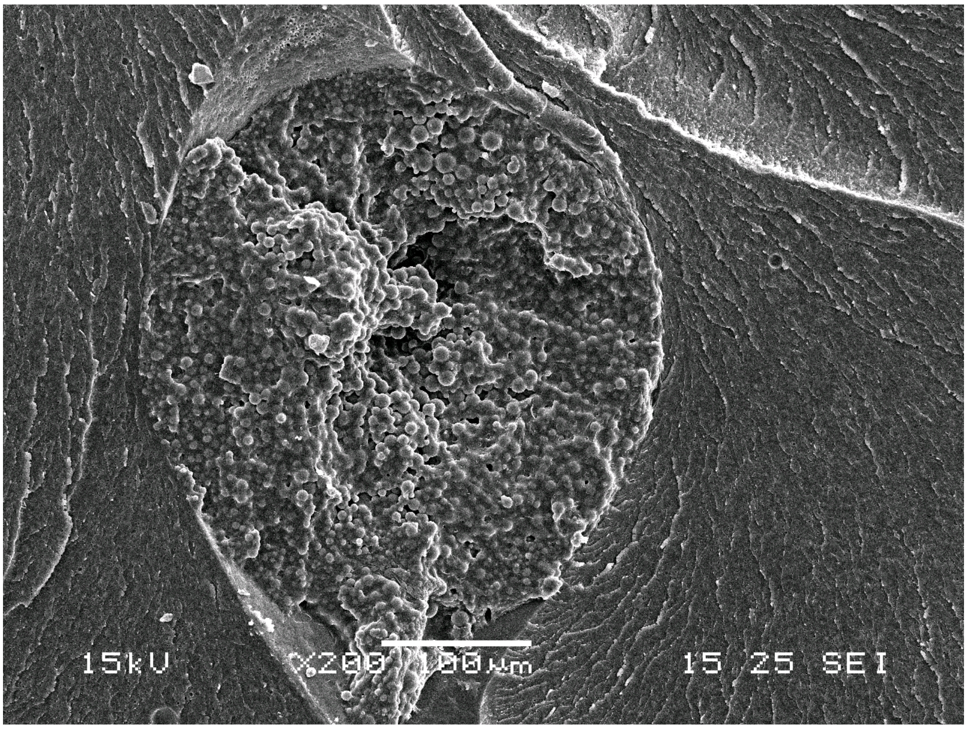

(C)

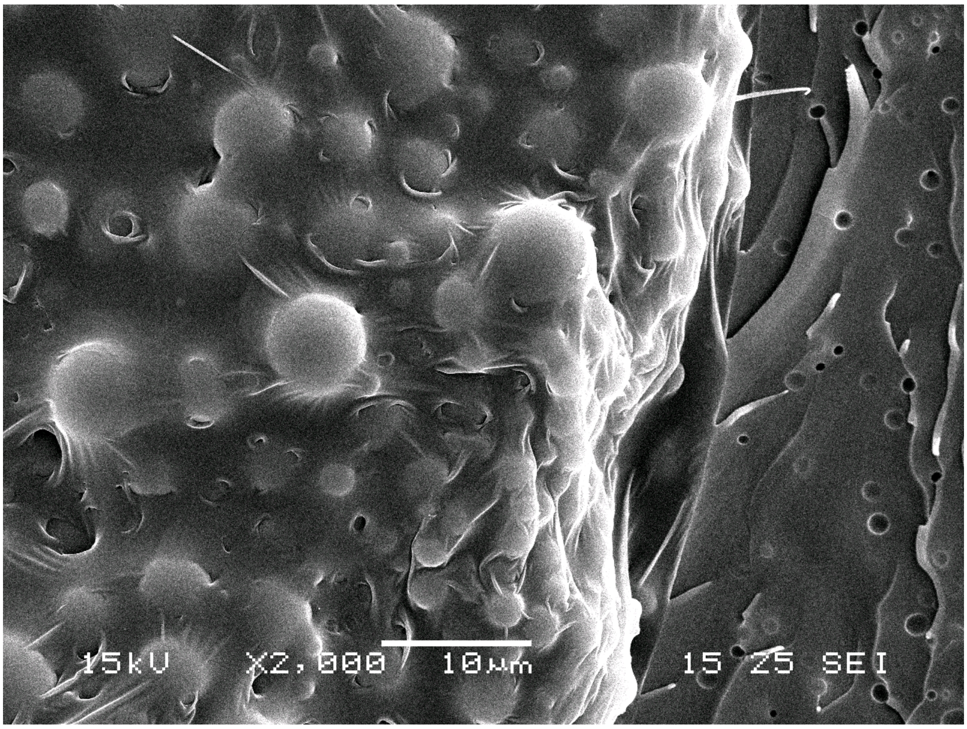

(D)

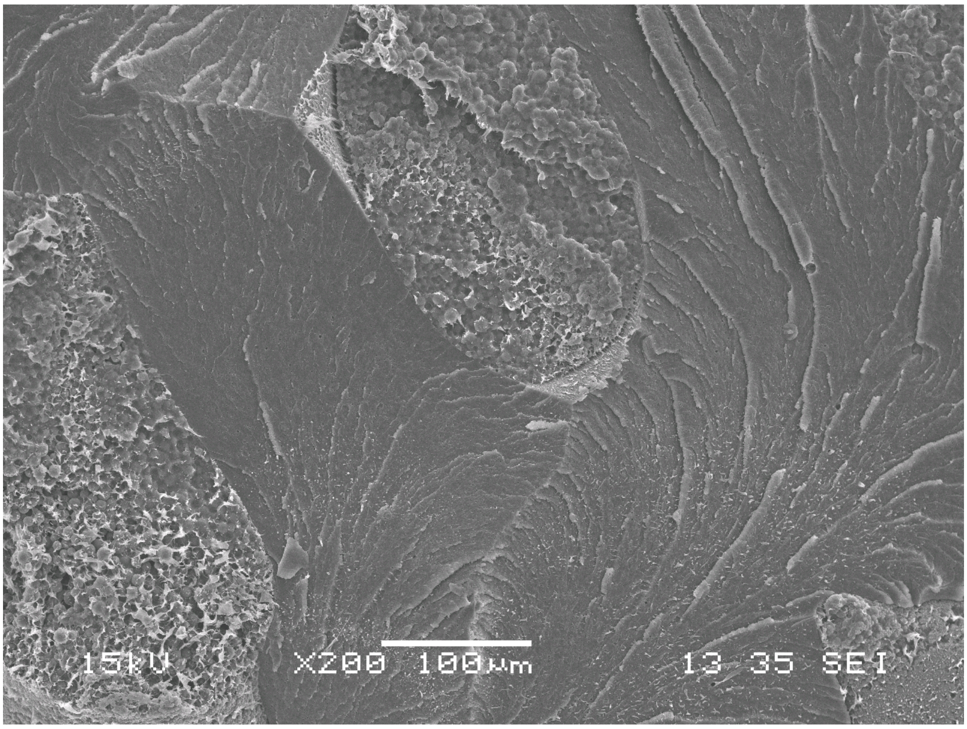

(E)

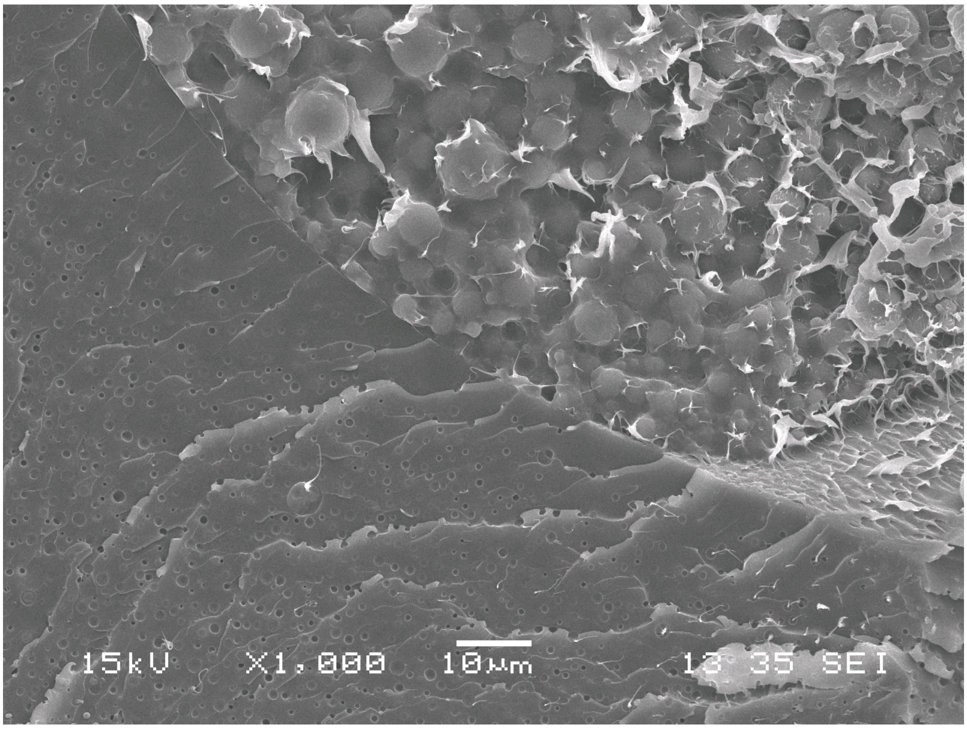

(F)

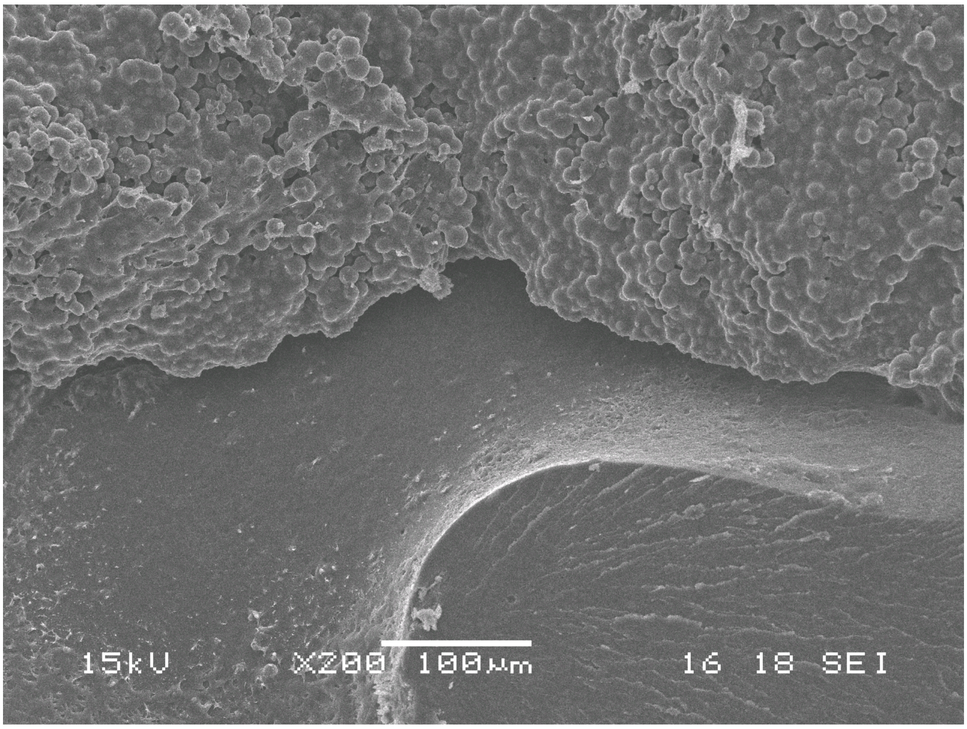

(G)

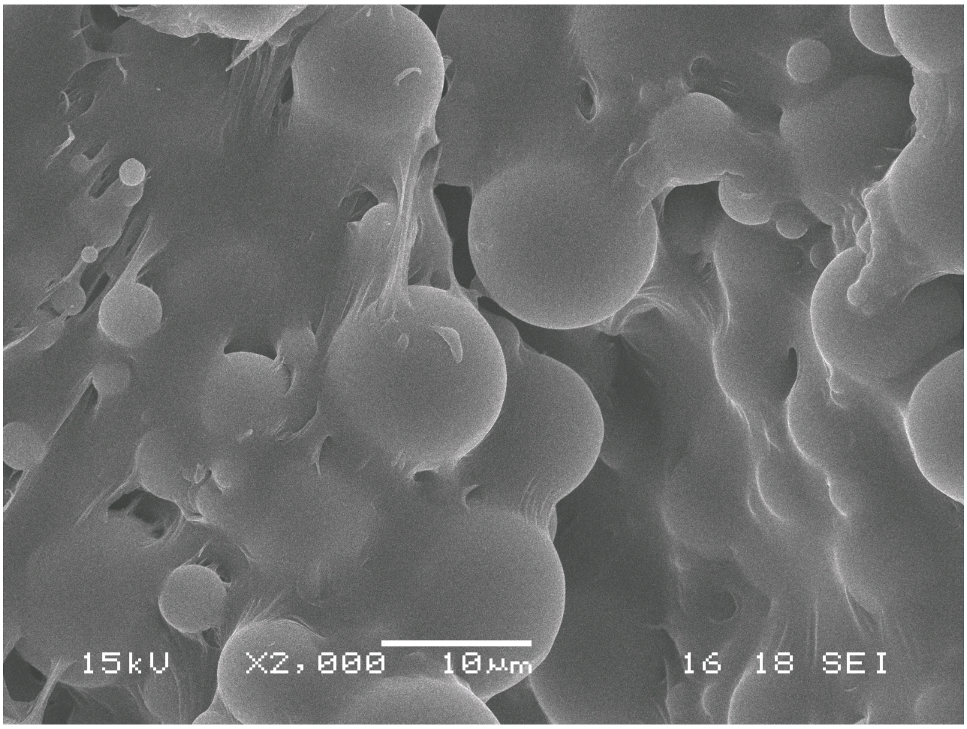

(H)

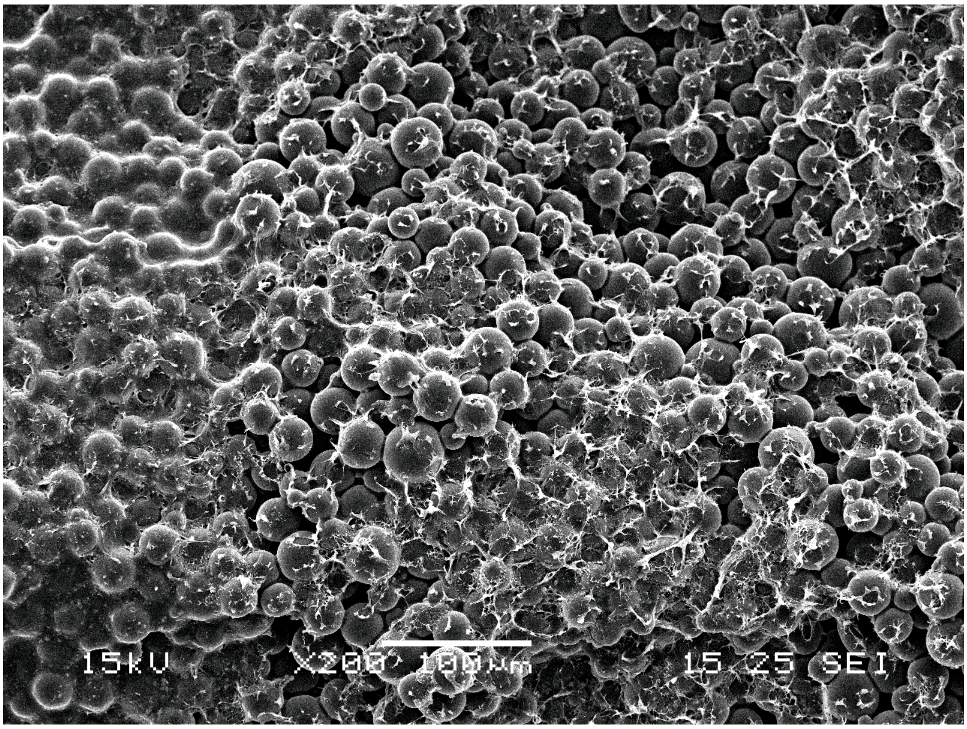

(I)

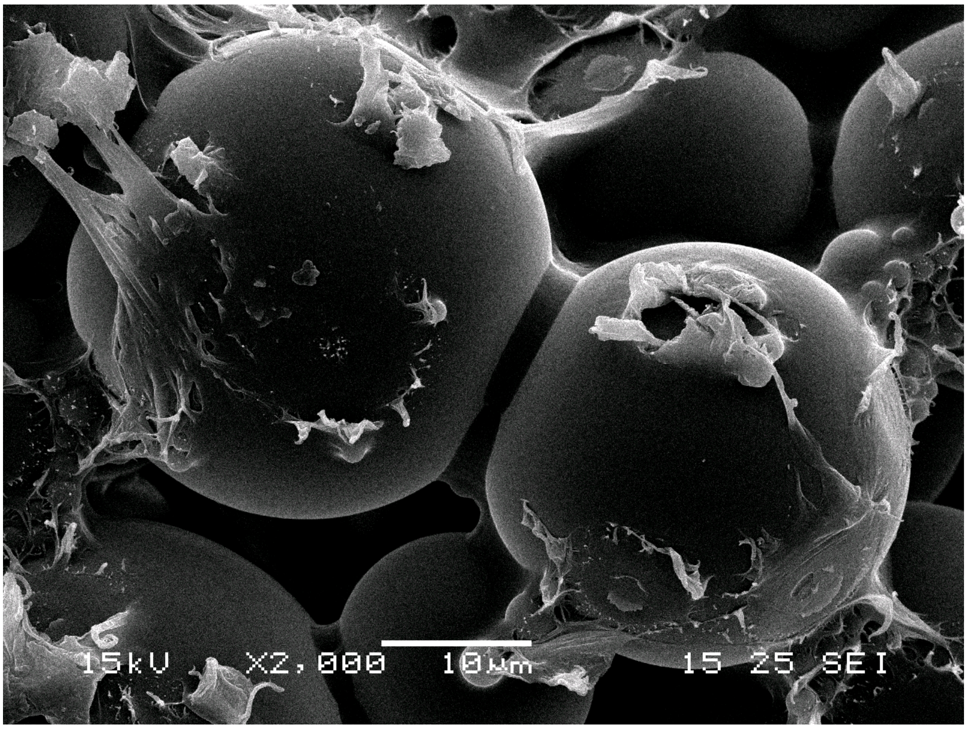

(J)

Figure S3. Original version of Figure 15 in main text: (A) T_{cur} = 200 °C, 22vol.%; (B) T_{cur} = 200 °C, 22vol.%; (C) T_{cur} = 200 °C, 23vol.%; (D) T_{cur} = 200 °C, 23vol.%; (E) T_{cur} = 200 °C, 24vol.%; (F) T_{cur} = 200 °C, 24vol.%; (G) T_{cur} = 200 °C, 25vol.%; (H) T_{cur} = 200 °C, 25vol.%; (I) T_{cur} = 200 °C, 26vol.%; (J) T_{cur} = 200 °C, 26vol.%.

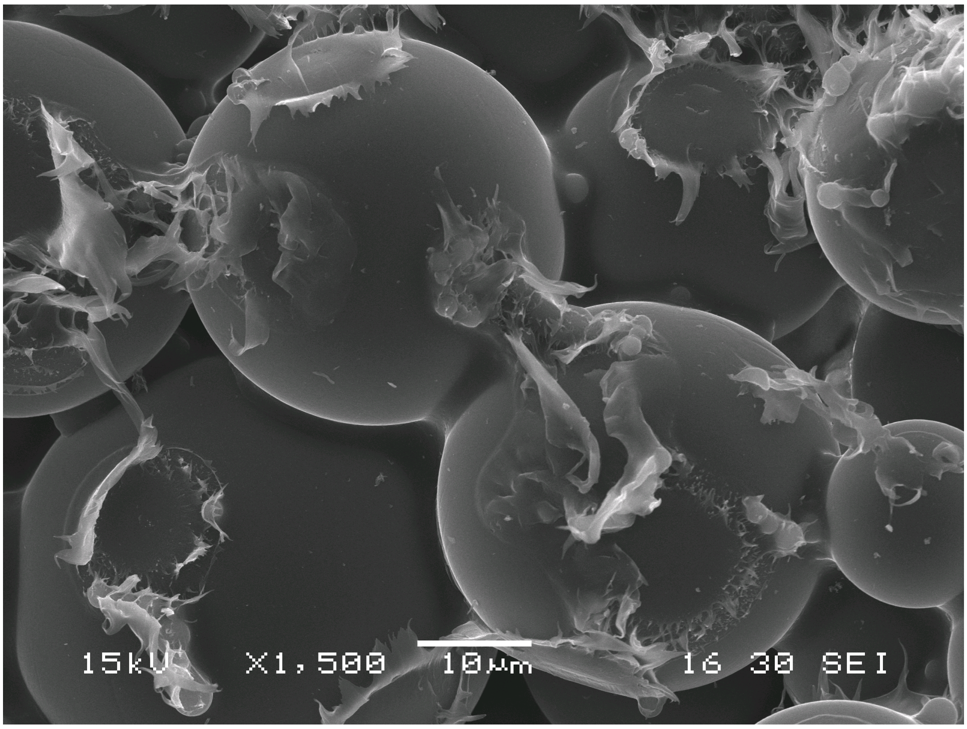

(A)

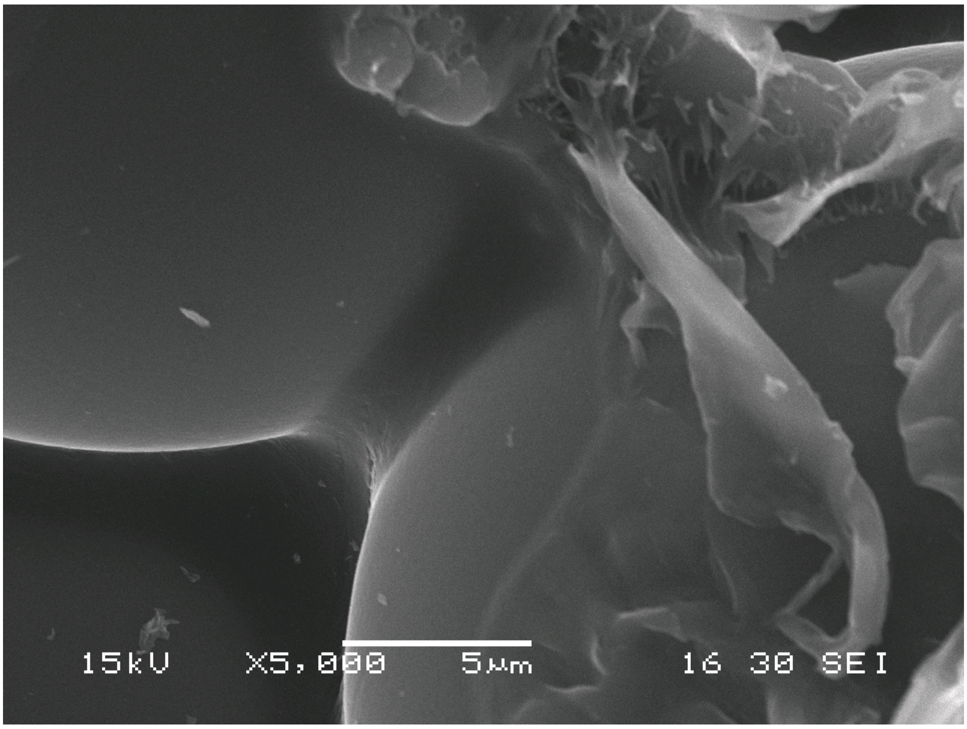

(B)

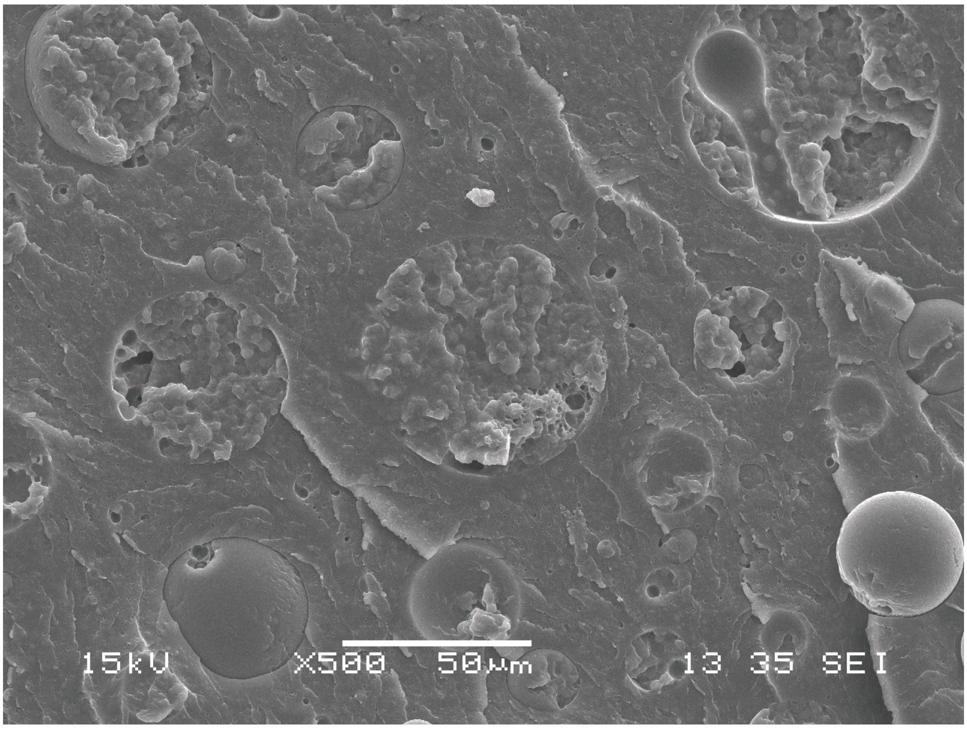

(C)

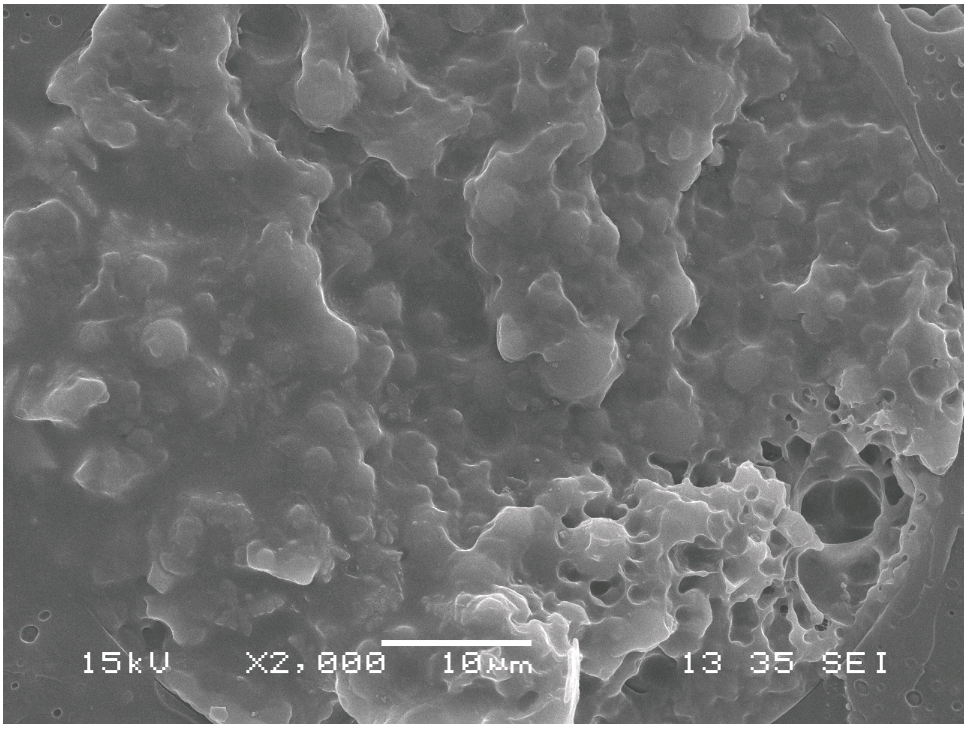

(D)

Figure S4. Original version of Figure 17 in main text: (A) T_{cur} = 200 °C, 26vol.%; (B) T_{cur} = 200 °C, 26vol.%; (C) T_{cur} = 200 °C, 22vol.%; (D) T_{cur} = 200 °C, 22vol.%.

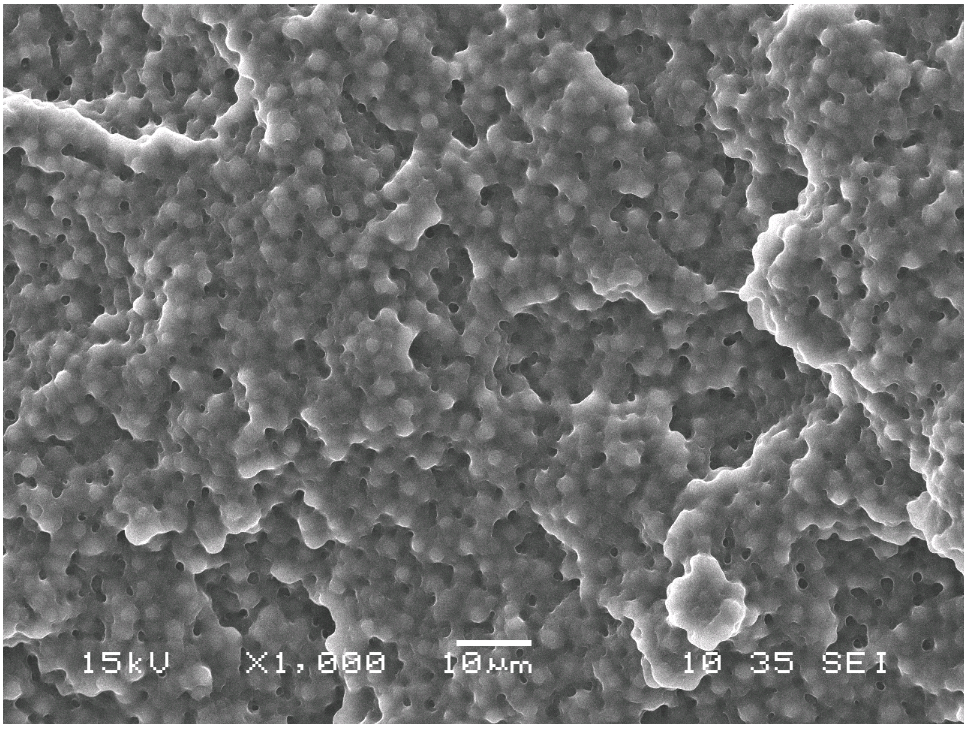

(A)

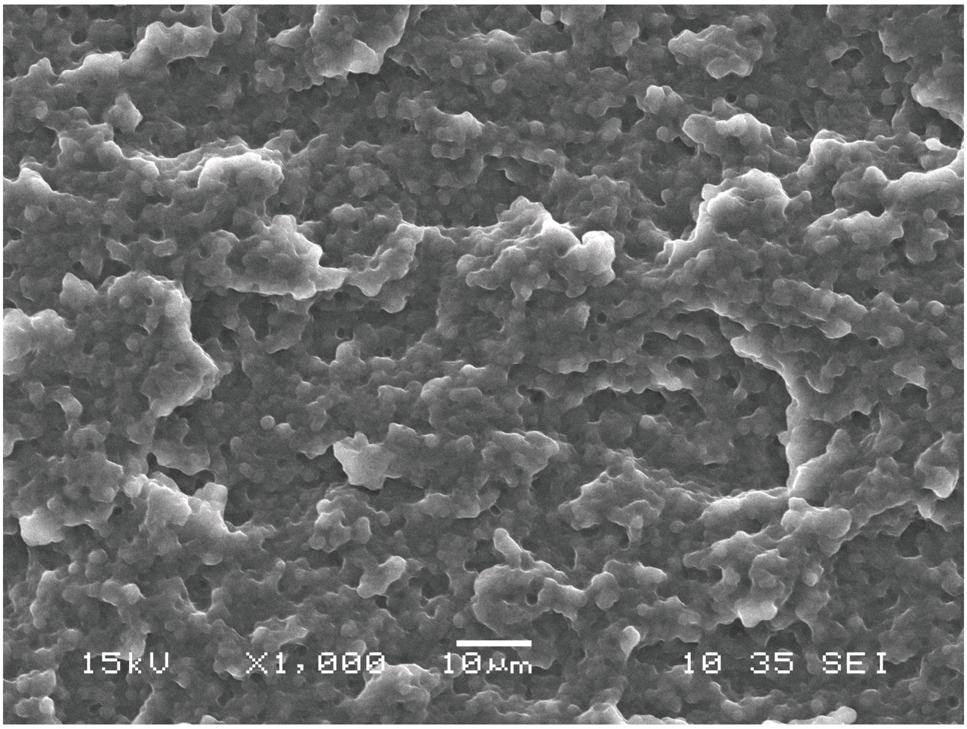

(B)

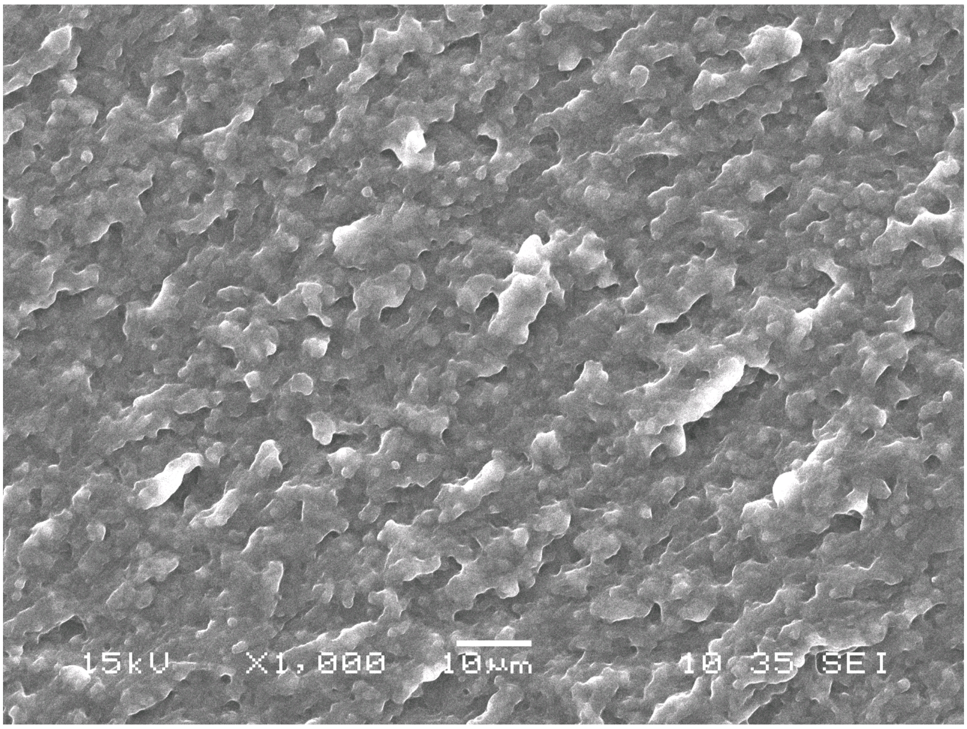

(C)

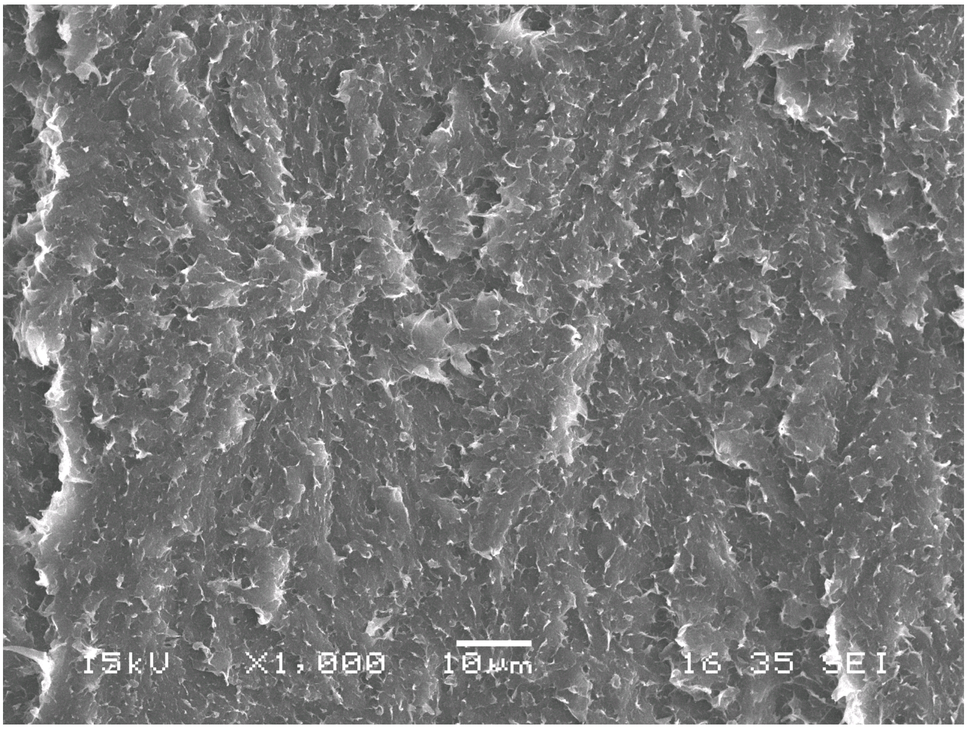

(D)

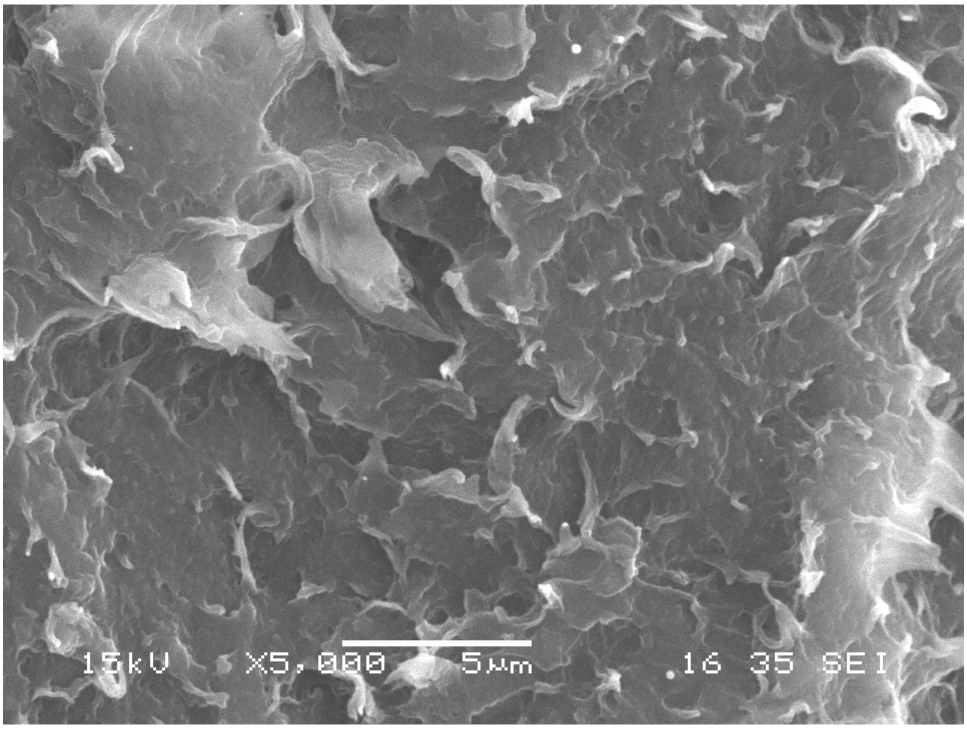

(E)

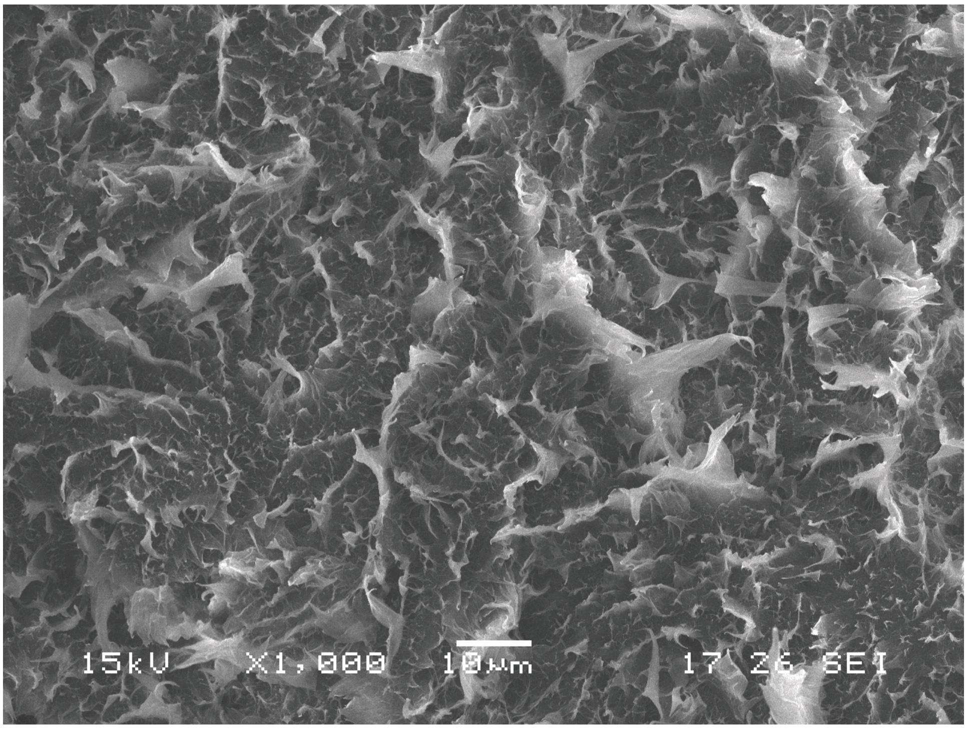

(F)

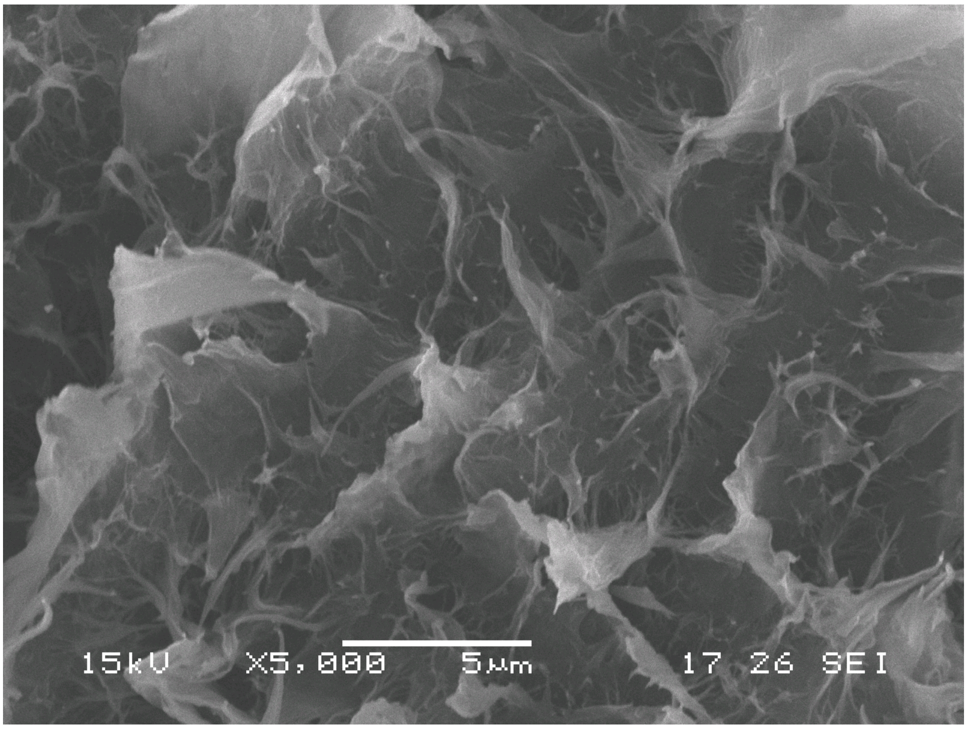

(G)

Figure S5. Original version of Figure 21 in main text: (A) T_{cur} = 200 °C, 50vol.%; (B) T_{cur} = 200 °C, 60vol.%; (C) T_{cur} = 200 °C, 70vol.%; (D) T_{cur} = 200 °C, 80vol.%; (E) T_{cur} = 200 °C, 80vol.%; (F)T_{cur} = 200 °C, 90vol.%; (G) T_{cur} = 200 °C, 90vol.%.

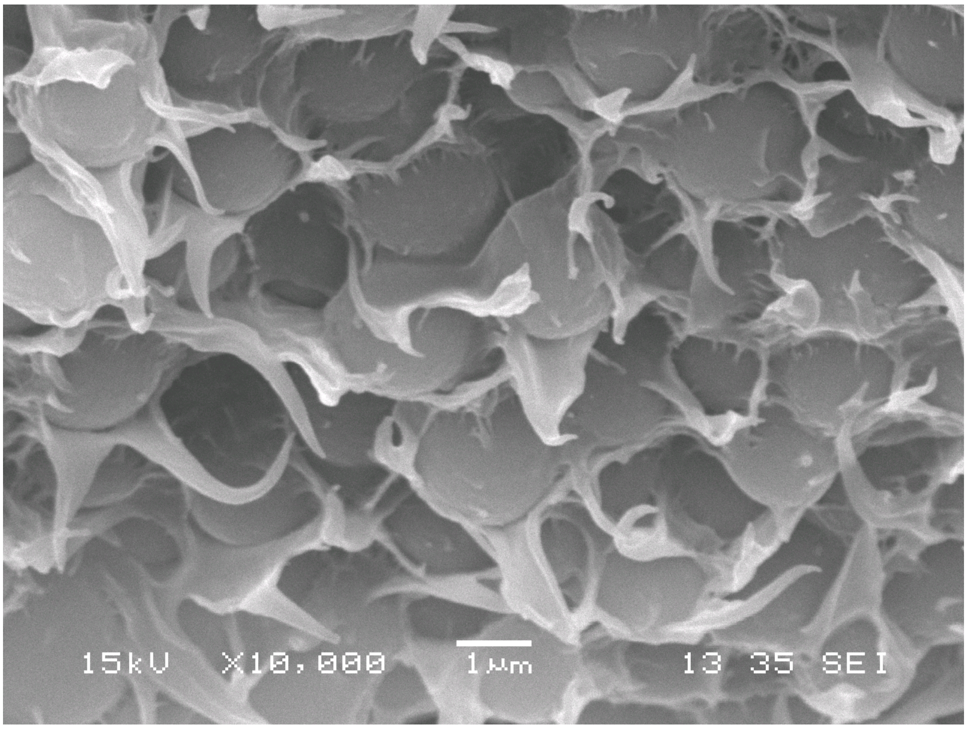

(A)

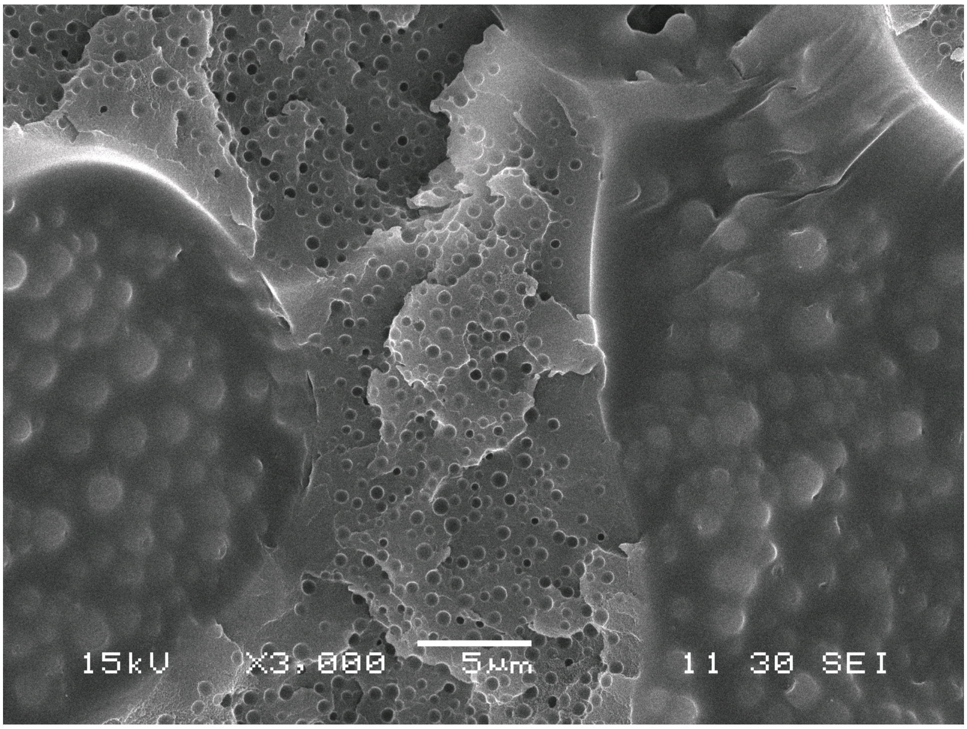

(B)

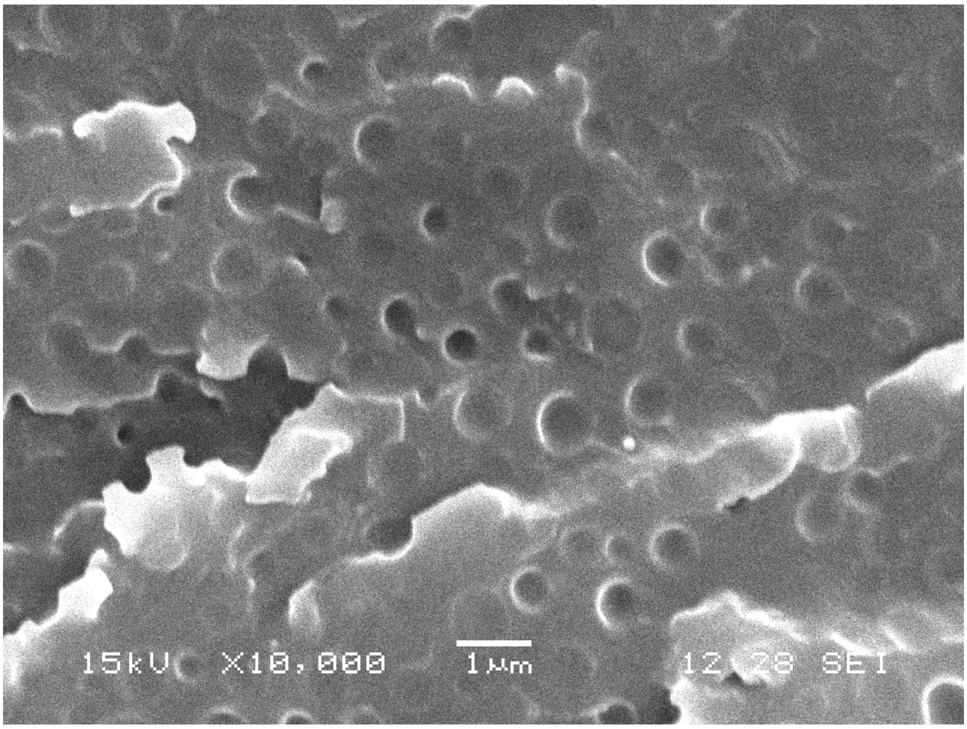

(C)

Figure S6. Original version of Figure 23 in main text: (A) T_{cur} = 150 °C, 38vol.%; (B) T_{cur} = 150 °C, 28vol.%; (C) T_{cur} = 150 °C, 23vol.%.
